# Supplementary material for: The influence of perceived medical risks and psychosocial concerns on photoprotection behaviours among adults with xeroderma pigmentosum: a qualitative interview study in the UK
Source: BMJ Open. 2019 Feb 5;9(2):e024445. doi: 10.1136/bmjopen-2018-024445 (PMC6377541; doi:10.1136/bmjopen-2018-024445)
Supplement: Supplementary file 1 [file bmjopen-2018-024445supp001.pdf]

Supplementary Table 1: Topics and examples of open questions from interview guide

---

Introduction

Explanation that this is the first time research has been done in XP from the patients perspective and we want to learn more about the day to day experiences and challenges of living with XP from your personal point of view. We are having this conversation to find out what it is like living with XP. We are interested in your views and understanding XP from your perspective. There are no right or wrong answers.

The interview is confidential and when we write up the study any quotations that we use will be anonymous and will not be linked to your name. The staff at the XP clinic will not hear your interview and will not know what you have said.

Taking part in the interview is voluntary - you can stop at any time. If you want to stop just let me know. If you want to take a break, just let me know. You can take your time to think about what you want to say – there is no rush.

In the information leaflet we explained that we would like to record the conversation. Is this still OK with you? This is to help us with the analysis.

Topics and examples of questions

1. Participants personal story of their XP
When were you first aware of having a skin problem? How was it diagnosed? What was your understanding of XP at that time?
What do you think are the risks of XP for you? How much does this bother you?
For how long have you been attending the specialist clinic at St Thomas' Hospital? Has this been helpful?

2. General views of photoprotection
How important is photoprotection for you? Do you think it is important in all seasons including winter and on cloudy days?
What are any particular downsides of photoprotection for you?

3. Photoprotection practices
How important is sunscreen for you? How regularly do you use sunscreen?
Do you go out without using? Are there any problems with it?

Do you make special efforts to wear clothing to cover up including gloves, long sleeves, long trousers and hats or hoodie? Glasses? Is this protection easy or are there problems with having to cover up like this?

Do you wear a visor? Is it comfortable? How important to use? If not wear currently -have you ever worn one? Why did you stop?

Do you avoid going out when sunny? How does this restrict your life?
Do you do any UV protection when inside?
Have there been any changes in your photoprotection over time?

4. **Psychosocial burden of XP/photoprotection**

What are the main ways having XP affects your activities, such as work/school/leisure activities?

What are your biggest worries about having XP?
Does having XP affect you emotionally?

5. Support by family and friends
Do other people understand/support you? In what ways? How important/helpful is this?

Do you worry about what others think about you?
Do you think others respond to you differently because of your XP?
Have you had any experiences that have made you feel this way? How do you cope with this?

Supplementary Table 2: **Response to photoprotection by age and gender**

	Study no.	Age (yrs)	Gender (M/F)
‘Dominated’	7	27	M
	13	28	M
	14	21	M
	22	26	M
‘Resistant’	3	37	M
	4	36	M
	5	26	F
	6	20	M
	10	39	M
	12	21	M
	15	34	M
	18	31	F
	20	55	M
	23	55	M
	24	28	F
‘Integrated’	1	45	F
	2	35	F
	8	63	M
	9	38	M
	11	16	M
	16	63	F
	17	62	F
	19	18	M
	21	62	M
	25	55	F
