# Supplementary material for: The influence of perceived medical risks and psychosocial concerns on photoprotection behaviours among adults with xeroderma pigmentosum: a qualitative interview study in the UK
Source: BMJ Open. 2019 Feb 5;9(2):e024445. doi: 10.1136/bmjopen-2018-024445 (PMC6377541; doi:10.1136/bmjopen-2018-024445)
Supplement: Author's manuscript [file bmjopen-2018-024445.draft_revisions.pdf]

BMJ Open is committed to open peer review. As part of this commitment we make the peer review history of every article we publish publicly available.

When an article is published we post the peer reviewers' comments and the authors' responses online. We also post the versions of the paper that were used during peer review. These are the versions that the peer review comments apply to.

The versions of the paper that follow are the versions that were submitted during the peer review process. They are not the versions of record or the final published versions. They should not be cited or distributed as the published version of this manuscript.

BMJ Open is an open access journal and the full, final, typeset and author-corrected version of record of the manuscript is available on our site with no access controls, subscription charges or pay-per-view fees (http://bmjopen.bmj.com).

If you have any questions on BMJ Open's open peer review process please email info.bmjopen@bmj.com

BMJ Open

Influence of perceived medical risks and psychosocial concerns on photoprotection behaviours among adults with Xeroderma Pigmentosum: Qualitative interview study in the UK

Journal:	*BMJ Open*
Manuscript ID	bmjopen-2018-024445
Article Type:	Research
Date Submitted by the Author:	25-May-2018
Complete List of Authors:	Morgan, Myfanwy; King's College London, Institute of Pharmaceutical Science<br>Anderson, Rebecca; King's College London, Institute of Pharmaceutical Science<br>Walburn, Jessica; King's College London, Institute of Pharmaceutical Science<br>Weinman, John; Kings College London, Institute of Pharmaceutical Sciences & Institute of Psychiatry<br>Sarkany, Robert ; Guy's and St Thomas' NHS Foundation Trust, St John's Institute of Dermatology
Keywords:	Adult dermatology < DERMATOLOGY, Adherence, Health Behaviours, Photoprotection, QUALITATIVE RESEARCH

SCHOLARONE™
Manuscripts

For peer review only

Influence of perceived medical risks and psychosocial concerns on photoprotection behaviours among adults with Xeroderma Pigmentosum: Qualitative interview study in the UK

Myfanwy Morgan,[1] Rebecca Anderson,[1] Jessica Walburn,[1] John Weinman,[1]

[1] Institute of Pharmaceutical Science, King's College London, UK

Robert Sarkany. [2]

[2] National Xeroderma Pigmentosum Service, Guy's and St.Thomas' NHS Foundation Trust, London, UK

Text = 5933 words

Corresponding author: Prof Myfanwy Morgan

Institute of Pharmaceutical Science, King's College London,
152 Stamford Street, London SE1 9NH

Email: myfanwy.morgan@kcl.ac.uk

For peer review only - http://bmjopen.bmj.com/site/about/guidelines.xhtml

Key words: adherence, dermatology, photoprotection, appearance, qualitative methods.

Abstract

Objectives: To examine photoprotection practices by people with a rare skin disease, Xeroderma Pigmentosum (XP), and the medical and psychosocial influences on their adherence.

Design: Inductive cross-sectional qualitative study to contribute to a theoretically based framework for developing individualized behavior change interventions.

Setting: Home interviews with adults who attend the UK National XP service in London.

Methods: Semi-structured interviews conducted between February and June 2016. Interviews audio-recorded and transcribed verbatim with a Framework analysis that included techniques of charting and mapping to examine relationships and facilitate comparison across cases. The credibility of emergent groups was assessed by the PPI group and through clinical data.

Participants: 25 adults, 17 male and 8 female, aged 16-63 years attending the UK National XP service. Eighteen lived outside London.

Results: Awareness of risks of UVR and knowledge of photoprotection were high but there was considerable variation in behaviours. Three types of response were identified:1) *'dominated'* by planning and routines to achieve a high level of photoprotection and reduce clinical risks with significant activity restrictions and psychosocial impacts; 2) '*resistant'* to photoprotection with significant gaps in practices reflecting immediate concerns of avoiding an illness identity and having a normal life; 3) photoprotection *'integrated*' with individual's life with little psychosocial impact. These responses were influenced by illness, personal and contextual factors including age, life stage and social support with only the 'integrated' group achieving an equilibrium between perceived 'necessity' and 'concerns'.

Conclusions: There were significant variations in photoprotection reflecting perceived risks of UVR and priority given to the immediate psychosocial impacts of photoprotection and activity restrictions. The study is the first to examine photoprotection behaviours by people with XP and will inform a series of individualized behavioural interventions that may also be applicable to other conditions with high risks of UVR related skin cancer.

298 words

For peer review only - http://bmjopen.bmj.com/site/about/guidelines.xhtml

Article Summary

Strengths and limitations of this study

. The first study to examine the reasons for non-adherence with photoprotection among people with xeroderma pigmentosum (XP)

. identifies three forms of response to photoprotection ('dominated', 'resistant' and 'integrated') characterized by a differing balance between perceptions of medical risks and psychosocial concerns.

. The study is part of a mixed methods programme of research to inform individualized behavior change interventions to reduce UVR exposure.

. The PPI group and the clinical team contributed to interpretation of data and establishing credibility of the emergent categories of behavioural responses.

. It was not feasible to undertake follow-up interviews to the influences on transitions between modes of response or to undertake respondent validation given the already significant demands of the research programme on a small sample of people with this rare disease.

For peer review only - http://bmjopen.bmj.com/site/about/guidelines.xhtml

Introduction

Globally the prevalence of chronic disease is increasing but adherence with recommended therapies remains persistently low; on average 50% of all patients with long-term therapy for chronic disease in developed countries are non-adherent with medication[1] and 50-80% for behavioural changes.[2] Both forms of non-adherence increase risks of morbidity and mortality with implications for demands on health services and costs of care.[3]

Research conducted to explain high levels of non-adherence with medical advice has identified a range of illness related, psychosocial and social structural influences.[4,5] However, very few studies have examined the determinants of non-adherence among people with a rare disease, defined by the European Union as conditions that affect fewer than 5 in 10,000 people in the general population (Cooper and Clatworthy, 2014). Rare diseases in totality are however not so rare, with over 6000 rare diseases identified accounting for 1 in 2000 people in the European Union and 1 in 1500 in the United States.[6] Moreover only about 5% have a medical curative treatment with most managed through lifestyle changes.[6]

This study focuses on adherence with recommended lifestyle changes among patients with Xeroderma Pigmentosum (XP), a rare disease caused by mutations in any of eight genes, resulting in defective repair of ultraviolet radiation (UVR) induced DNA damage.[7] The incidence is 2.3 per million live births in Western Europe.[8] Patients may develop skin cancers from childhood onwards, with an estimated 2,000 and 10,000 fold increased risk of melanoma and non-melanoma skin cancers respectively that particularly affect the face, together with increased risks of ocular problems and neurodegeneration. [9] About half the patients comprise 'burners' who suffer abnormal and severe sunburn reactions and blistering as measured by an XP sunburn score and the highest risks of neurological degeneration, whereas 'non-burners' have an entirely normal sunburn response but the highest risks of skin cancers at an early age and ocular problems.[10]

There is currently no curative treatment for XP. Improving life expectancy and reducing risks of skin cancers and eye disease therefore depends on rigorous long-term UVR

For peer review only - http://bmjopen.bmj.com/site/about/guidelines.xhtml

protection. This involves regular use of sunscreen (SPF 50+), covering arms and legs with layers of clothing and wearing gloves, a UVR protective visor or other head/face covering, UVR protective glasses and attaching UVR protective film to windows. [11]

Research to date has shown that sun protective knowledge is high among general populations[12] and among high risk groups [13,14] but has not adequately explained low adherence. Furthermore no research has focused specifically on people with XP, although expert clinical opinion suggests these patients may vary widely in the degree to which they photoprotect.[15]

The current study forms one component of a five year NIHR funded programme of research that aims to develop and evaluate interventions to increase adherence with photoprotection and thus reduce measured UVR exposure among people with XP.[16] The programme is guided by a systematic theoretically based framework to inform behavior change interventions.[17] The first phase requires a comprehensive understanding of photoprotection behaviours based on mixed methods research to inform the development of individualized interventions.[16] This research comprised a cross-sectional survey to assess potential clinical, socio-demographic and behavioural correlates of photoprotection behavior, an N-of-1 study using daily diaries to record photoprotection activities, and ratings of potential psychosocial predictors and measurement of UVR dose to the face.[16] These quantitative studies were complemented by this more in-depth qualitative study to examine the 'why', 'what'' and how' questions to inform the development of personalised interventions in phase 2.

.

Aims

To elicit individuals' personal accounts of their photoprotection behaviours and to explore the influence of perceptions of UVR risks, psychosocial and contextual influences on adherence.

Methods

Design: Inductive qualitative study based on semi-structured interviews with analysis employing a Framework approach.[18]

For peer review only - http://bmjopen.bmj.com/site/about/guidelines.xhtml

The SRQR guidelines were followed.[19] (Supplementary Table 1)

Setting: The UK National XP service was set up in April 2010 at Guy's and St Thomas' Hospitals to provide a co-ordinated specialist service to achieve better outcomes. It currently has 90 adult patients diagnosed with XP on the register who were referred for diagnosis and most attend annual checks and follow up care provided by the multi-disciplinary team.

Patient and public involvement: Six PPI members were recruited but two parents dropped out as their child was having severe health problems.The remaining four PPI members (1 patient, 2 parents, 1 teacher) participated in two discussion sessions to consider the qualitative findings. One parent was a co-investigator and contributed with the research team throughout from the initial application.

All study participants were sent a brief newsletter that provided study feedback. They were also invited to a thank you event that included informal discussion of the results. Further dissemination will occur following the trial phase of the research.

Recruitment: Participants recruited through the UK National specialist XP service in London. Eligible adults were identified from the clinic register and selected if satisfying the following criteria: aged 16 years and over, confirmed diagnosis of XP (reduced unscheduled DNA repair in fibroblast DNA repair assay), no neurodegeneration, adequate English to participate in an interview, and had not opted out of research.

The research nurse sent study information to the 38 eligible patients and two weeks later followed up with a phone call to enquire whether they were interested in participating. Seven could not be contacted and six declined due to time or not wishing to talk about XP.

Data collection: Twenty-five one-to-one interviews interviews were conducted. They were held in a private room in the patient's home with the exception of one phone interview for logistical reasons. Interviews were carried out between early February and June 2016 by a Research Fellow (JWal) and Research Assistant (RA) who are both trained in health psychology and experienced interviewers but had not previously studied

For peer review only - http://bmjopen.bmj.com/site/about/guidelines.xhtml

XP. Participants were aware that the interviewer was a non-clinical researcher employed at XXx College London to conduct the research. An interviewer (female) visited the patient's home with the research nurse who had made the initial contact with participants to provide continuity. She introduced the interviewer and obtained informed consent but was not present for the interview and when required looked after participants' children.

Interviews were structured by a topic guide and took the form of a guided conversation (Supplementary Table 2). They began with a brief reminder of the aims of the study and then focused on aspects of the patients' personal story to identify individuals' early experience of XP. This was followed by eliciting their current views and experiences of photoprotection, the psychosocial impacts of photoprotection and support by family and friends. Topics were discussed in any order according to the flow of the conversation and responses probed and clarified as necessary. Interviews were audio recorded with permission and mainly took 30-60 minutes. Participants were generally willing to provide detailed accounts of their photoprotection behaviours which appeared to be facilitated by taking the form of a conversation held in their home and by their own positive vews of the clinic and their support for the research. However reflective notes made following the interview identified a few participants as becoming upset with thinking about XP and some preferred not to discuss earlier stages and only to focus on the present but were all happy to continue the interview.

Data saturation was achieved with the 25 interviews with no new beliefs or explanations identified in the final interviews.

Analysis: This occurred alongside data collection and employed a Framework approach. [18] This goes beyond a thematic analysis and involves methods for developing explanations and classifications that are geared towards producing practice-oriented findings and was thus appropriate for a study to inform behavioural change interventions.

Tapes were transcribed verbatim, checked for accuracy and corrected transcripts entered into QSR NVivo11 software. Transcripts and fieldnotes were initially read to identify key themes. Coding was then undertaken with the initial coding framework adapted as new items arose. One author (RA) had a major role in coding (i.e.' indexing')

For peer review only - http://bmjopen.bmj.com/site/about/guidelines.xhtml

with two other authors (JWal, MM) repeating and reviewing the coding which was discussed to achieve consensus, with all three reading and familiar with all the transcripts Analysis included comparisons within and across cases which was assisted by 'charting' that involves summarizing data by thematic content for each case to allow relationships between codes to be identified.[20] These charts were further examined through a process of 'mapping and interpretation' leading to emerging ideas and relationships. Preliminary behavioural classifications were tested through further analysis to identify the fit for individual cases and refinements were introduced as required. This iterative process finally led to a 3-fold categorization of photoprotection behaviours based on perceptions of risks of UVR and the psychosocial impacts of photoprotection, with some variation noted within these broad categories that was influenced particularly by age and whether protection began in childhood.

Credibility and triangulation.

The credibility of the emergent categories in terms of their congruence with reality forms a key aspect of validity in qualitative research.[21] This was informed by the patient and public involvement (PPI) group who participated in two sessions to consider the qualitative findings. Two clinical discussion groups were also held with a total of eleven clinical specialists who undertake the patients' annual check to identify their clinical views of the emergent categories and awareness of the reported psychosocial impacts and drivers. The level of adherence with photoprotection of our three patient categories was also examined based on prior clinic reviews conducted by specialist nurses using a 13-item measure of photoprotection behaviours.[22]

Results

Characteristics of participants: Twenty-five adults were interviewed. Participants were aged 16-63 years and 18 lived outside London. Seventeen were male and eight female reflecting the adult clinic caseload (26 males and 17 females). Eight were European (mainly white British), eight of Pakistani ethnicity and seven mainly of Middle Eastern, Indian or Bangladeshi background reflecting the relatively high prevalence of XP in Pakistan and the Middle East.[7]

For peer review only - http://bmjopen.bmj.com/site/about/guidelines.xhtml

Seventeen participants were defined as 'non-burners' (i.e. normal sunburn response) and eight as 'burners' (i.e. abnormal sunburn response to minimal sun exposure).[10] Those categorized as 'burners' had all begun some form of photoprotection in childhood following recognition that they burned easily, whereas half the 'non-burners' had begun photoprotection after 10 years of age. Self-reported clinical diagnosis occurred more than 5 years ago for all but five participants and all currently attended the specialist clinic annually. The 'burners' had experienced reddening and blistering, pigmentation changes particularly freckles and benign lesions. In contrast nearly all the 'non-burners' had experienced removal of one or more cancerous lesions.

Knowledge of risks and photoprotection. Participants were all aware that XP is a long-term condition with risks of skin damage and both benign and cancerous lesions. They were also aware of requirements for photoprotection and described their knowledge as increasing over time through greater experience of the condition and attending the specialist clinic. For example, some participants initially thought only the face is affected by UV exposure and were therefore surprised to require lesions removed from their neck, arms or legs, As participant 28 (female, age 28) explained:

''….so my neck, so then I'll wear a hat and I'll wear sun cream more often, and I'll wear my sunglasses. Then there's bits on your arms, so I'll start wearing long sleeve t-shirts. And then this happened on my leg…..but now I'm like no, I'm going to buy leggings and I'm going to wear leggings with long sleeve tops.'

All participants engaged in some photoprotection but when asked why they did not undertake particular aspects often found this difficult to answer, although further discussion identified a range of positive and negative factors that appeared to influenced how they interpreted and balanced medical and psychosocial priorities. Their explanations led us to identify three main types of response to photoprotection: '*dominated*' by photoprotection, '*resistant'* to photoprotection and *integrated*' photoprotection (Figure 1).

Figure 1: Behavioural responses to photoprotection among people with XP

Responses to photoprotection

For peer review only - http://bmjopen.bmj.com/site/about/guidelines.xhtml

1. Dominated by demands of photoprotection. This group comprised four men (one 'burner' and 3 'non-burners') aged between 21 and 28 years who all described a very high level of photoprotection. Their major motivation for undertaking rigorous photoprotection was their concerns about the harmful effects of UVR exposure. The three non-burners feared cancerous lesions and a reduced length of life, whereas the major worry for the one 'burner' (Participant 15 age 22) was having very painful and unpleasant sunburn reactions. However for all these participants an immediate concern was the effects of UVR on their appearance due to freckling and other pigmentation changes including facial scars following surgery. For example, Participant 14 a non-burner (male, age 21) explained, '*If you take it purely objectively, I don't think I'm as unfortunate as some skin patients are (effects on appearance). But you still worry about it. Yeah, that's a main worry.'*

People 'dominated' by photoprotection had begun sun protection in childhood but described adjusting to it better as they got older and often did not want to think back to earlier stages, describing this as '*too long ago.'* However participant 07 (male, age 27) explained that at school and college he never used his sunscreen '*because I wanted to feel normal, that's why...I didn't want any questions, or any remarks from friends or people that I didn't know, and I did not want to look abnormal to anyone else.'* This changed when he had his first surgery to remove a basal cell carcinoma which he described as a '*reality check'* and '*wake-up call' as 'I was putting myself in harms' way for no reason…just destroying my own skin.*' From that time he gave considerable emphasis to the risks of UVR and the need for rigorous photoprotection.

All four respondents described having UVR protective film on the windows of their home and car and used a UVR meter to assess risks and identify appropriate environments and locations. They also described well-established behavioural routines for photoprotection. This involved applying sunscreen whatever the season and wearing two or three layers of UV protective clothing, gloves and a hat or cap with a hoodie or a visor when outside during the day. A visor provides full cover of the face and head and is usually only worn by children. However a visor was worn by three people categorized as 'dominated'. Thdey described a visor as uncomfortable to wear, bulky to carry around and attracting stares and comments but users regarded a visor as necessary to achieve

For peer review only - http://bmjopen.bmj.com/site/about/guidelines.xhtml

sufficient protection and formed part of '*going out properly protected*.' The one participant in this group who did not wear a visor but otherwise engaged in rigorous photoprotection regarded a visor as also having negative psychosocial impacts:

'*..totally make me feel abnormal. Just wearing a hat, cap and glasses* all *the time, I've gotten people questioning like mad, so imagine wearing a visor.'* (Participant 7, male, 27yrs)

Participants 'dominated' by photoprotection explained how their practices had shifted from a deliberative action into a routine or 'habit' thar they *'do without thinking, it becomes part of your daily routine*.' As one participant observed,

'I think I'm motivated by keeping myself safe and I don't want to develop any skin cancers which is a problem for my condition. But really, rather than motivation it's something I do just because it's a deeply ingrained in me and because I've been doing it forever.' (Paricipant 13, male, age 28)

This high level of photoprotection carried considerable personal costs. These included restrictions on choice of job with risks of exposure to UVR forming an important consideration:

'It's affected the kind of jobs that I can look for. I only choose something that's inside in a safe environment and I really can't go for anything else.'. He now works as a shop assistant in a large supermarket (Participant 14, male, age 21). Others worked as a barber, in the family takeaway or as a health scientist rather than in their job of choice.

Participants also described missing out on social events because they did not want to wear UVR protection or did not regard it safe to go outside even with full protection. For example, participant 13 (male, age 28) explained,

'I make excuses about not going out with friends and prefer to be indoors between 11 and 4 when the sun is at its highest.'

If going out during the daytime was unavoidable they undertook considerable preparation. As participant 22 (male, age 26) explained, '*the only times I go out during the day is if I'm going from a car to a building or building to car, or if it's completely unavoidable*….' This decision in turn cues his usual routine that involves '*suncream, visor, everything*.' He described this as taking him *'……..about half an hour just to get*

For peer review only - http://bmjopen.bmj.com/site/about/guidelines.xhtml

ready because I've got to apply my sun cream, then I've got to be sure I haven't missed any areas. I also apply lip balm which is UV protected. Then once I've applied my sunscreen I apply my camouflage (to hide scars). I apply two camouflages.' He also explained, '*If I've got to be somewhere for 5pm I'll have to leave at 4.30pm which means I've got to cover up between (workplace) and the tube station. So I need the visor that day.*'

This group referred to the psychological costs of a high level of photoprotection in terms of being viewed as different or not 'normal'. However they described no longer being upset by stares and questions about their clothing and if people continued to ask questions or make comments they now felt more confident and able to '*put them in their* place.' However, they still sometimes avoided situations that might lead to '*snide remarks'*, such as taking a taxi rather than a bus when school children were travelling.

Despite considerable support from family and friends this group described feeling emotionally distressed about the demands of photoprotection and its restrictions on their current life and activities. For two participants the impacts were quite severe. For example, participant 7 (male, age 27) explained, '*whatever I do XP is on my mind first'* and described XP as '*affecting me emotionally quite a bit as well.,,,,,It's just you get obsessed and get that depressing feeling. You can't do much in life.'* Similarly participant 22 (male, age 26) described '*feeling down'* about having XP and its effects on his life and described experiencing '*bouts of depression*' about three or four times a year. However these various adverse effects did not disrupt their high level of photoprotection given their fears of the potential clinical risks.

2. Resistant to photoprotection. This group comprised 11 participants, aged 20-55 years, with three 'burners' and eight 'non-burners'. All accepted their diagnosis of XP and were aware of the risks of UVR and required photoprotection practices. They therefore had the capability to photoprotect but acknowledged a lack of motivation and instead gave priority to their immediate desire '*to live a normal life'*.

For five people, aged 21-37 years, their lack of photoprotection routines largely reflected their desire to distance themselves from XP and be accepted as normal by others. For example, participant 24 (female, age 28) had experienced several surgeries to remove

For peer review only - http://bmjopen.bmj.com/site/about/guidelines.xhtml

skin cancers and benign lesions and was particularly concerned about surgery to her face. Nevertheless she described joining her friends for activities in the sun, not reapplying sunscreen and sometimes going out wearing shorts or summer tops, because as she explained:

'*I just want to be seen like everybody else'* and "*I think you can just let these things (photoprotection) become you and I don't want to be about XP. XP is a tiny part of me.'* She therefore sometimes thinks, '…*do you know what, I'm just going to get on with my life.'* She excused her behavior at interview by explaining that when out she 'shade hopped' and also appeared to gain reassurance from knowing that the clinic would identify and manage any problems.

Concern about feeling different and viewed by others as possessing an unacceptable difference was common in this group although their particular circumstances differed. For example, an Asian participant (no. 6, male, age 20 years) described freckles as highly unusual and abnormal on a person with Asian skin and reported that comments and questions by the Asian community made him feel '*like an outsider'* which corresponds with the notion of 'felt stigma' or feelings of shame associated with an unacceptable difference. [23] This response contributed to his stress and loneliness and to a reluctance to increase the obviousness of his condition through rigorous photoprotection.

This group of participants therefore described tensions between their desire to be viewed and participate as normal and worries about the effects of UVR without sufficient protection. For example, participant 18 (female, age 27) worries about cancer but often feels '*a bit left out'* as she can't go outside at lunch with colleagues. She also acknowledged that although she sometimes wears long sleeves in summer she does not usually wear a hat or reapply sunscreen which she regarded as greasy and staining. She explained that '*some days I don't bother with cream or covering up…..Sometimes I think screw it, just go out. Then I think, oh, sugar,….But there's consequences, just like oh my god.'* Similarly participant 12 (male, age 26) explained that he worries about life expectancy and appreciates the advice and support given by the clinic but still goes out and socializes in the summer'…*just to make myself feel better really, That's about it.*'

For peer review only - http://bmjopen.bmj.com/site/about/guidelines.xhtml

Concerns about being perceived and responded to in terms of XP led to concealing the condition by reduced photoprotection, the use of a tinted foundation with a lower SPF and reluctance to disclose their condition to others. Reminders to photoprotect by family members were often described as unhelpful as participant 12 explained, '*I just think it's a constant reminder that I'm different. So that's why I don't really like them (reminders).'* (male, age 21)

The other six people categorized as 'resistors' were mainly aged from their mid thirties to mid fifties and were not bothered about people knowing about their XP. However they had gaps in their photoprotection routines, often going outside without sunscreen. This appeared to reflect perceptions of low personal risks, especially if they had lived 20 or 30 years without major problems. There was also a view that life is uncertain and that exposure to UVR is just one of many risks they might encounter. They therefore preferred to *'live for today' and* have '*a happy life despite XP*.' As participant 20 (male, age 55) explained:

'I don't automatically put on sunscreen everyday even though I know I must….there's no downsides to sunscreen, so it's just time or not thinking about it or being too busy, or thinking no I can't be bothered.' He later explained, *'I would rather do things that give me pleasure in life rather than get overly* anxious *about the ransom to the possible outcomes of not taking enough care.*'

Some people referred to specific personal experiences as shaping these views, such as a close relative dying unexpectedly at a young age. Other reasons given for resisting rigorous photoprotection was a questioning of the effectiveness of sunscreen or of photoprotection generally, especially if they had lesions on parts of the body normally covered.

All participants in the 'resistant' category thus gave priority to leading a normal life but differed in whether the major influence on their behaviours arose from feelings of unacceptable differentness, or from perceptions of personal risk of UVR compared with other risks and demands of life.

3. Integrated photoprotection This category comprised ten people who represented the full age range from 16 to 63 years, with 4 'burners' and 6 'non-burners', All currently

For peer review only - http://bmjopen.bmj.com/site/about/guidelines.xhtml

accepted the need to undertake photoprotection, although a few people struggled initially as the demands of photoprotection made them feel like a 'sick' person. However, over time these practices had become *'automatic'*, '*habitual*' and *'just second nature'*. Photoprotection was therefore not currently viewed as a major practical or emotional burden leading to a 'non-normal' life and illness identity. Instead it was described as '*just a part of life … It's just habit now, a routine, what I do….It's just second nature I think for me' (Participant* 19, male, age 18) and as being '..*no hardship to do because I just do it as part of my daily routine*' (Participant 16, female, age 63). These participants generally felt comfortable and confident in their photoprotection routines and currently did not worry much about the risks of UVR.

This accommodation to photoprotection was described as occurring in one of two ways. A group of younger people diagnosed in childhood had spent most of their time indoors and now preferred this. For example, participant 19 (male, 18 yrs) had been diagnosed when 9 years old but began to protect much earlier. Currently his routine was to set off for work when dark at 6am and his father collected him later by car. Otherwise he was mainly in the house and preferred being indoors where he played with the computer. Participant 01 (female, aged 45) also described her need to keep out of the sun as fitting quite well with her life because she was at home most of the day looking after children.

Others emphasized how they accommodated the requirements of photoprotection without withdrawing indoors. For younger people this was often facilitated by friends or family members who accepted their need to avoid UVR and adjusted activities to ensure that they were not made to feel different or excluded.

'….especially when we're going out and there's a place that's not safe and then they all understand we probably need to move somewhere else and not do what everyone wants to do, which is to enjoy the sunlight.' (Participant 09, male, age 38)

Family members also organized activities when the sun was low, encouraged indoor sports and going on holiday in the UK rather than to hotter countries. Partners also often adapted their roles, as participant 10 (male, age 39) explained:

'I'm the one who would normally be staying in the car (with window film*)….and the missus would actually go into the shops, into the street to find whatever we need to get.'* ,,,,,,,,,,,,,, *'My wife tends to do the outdoor activities with the kids, and I do the indoors.'*

For peer review only - http://bmjopen.bmj.com/site/about/guidelines.xhtml

For older people their accommodation of photoprotection had often become easier through their long-standing experience of XP, as participant 16 (female, age 63 yrs) commented,

'Because I'm this great age now, I'm used to it….it's just part of my daily routine now…..the only time it impacts is if I'm invited to an outdoor event and either I don't go or wear a hat and stick to the shade….but that's the only time it impacts you. But I'm used to it now' (Laughter).

For people aged over 50 years the changes in what they expected and wished to do also reduced the personal social costs of photoprotection:

When younger I wanted to sunbathe and go on holidays with friends to hot countries but as the years went by I got used to it. Now it doesn't bother me.' (Participant 21, male, age 62)

Older people who had integrated photoprotection described rejecting any stigma associated with photoprotection, with negative views and comments viewed as *'other people's problem, not my problem'.* Although people who had integrated photoprotection into their lives were confident that their routines provided adequate personal protection they sometimes acknowledged scope for improvement and may not have been aware when their practices were not sufficiently protective.

Credibility and triangulation.

The varying behavioural response to photoprotection advice were presented and discussed with the four active members of the patient and public involvement team. The findings were endorsed with a minor revision suggested that was incorporated.

The two discussion groups with clinical specialists indicated that they regarded the three emergent categories as fitting with their clinical experience. They also viewed the psychosocial data as helpful in increasing their understanding of the range of meanings and contextual influences on photoprotection behaviours.

For peer review only - http://bmjopen.bmj.com/site/about/guidelines.xhtml

A 13 item measure that is routinely used by clinic nurses to assess adherence with photoprotection was checked following the classification into three groups and so did not influence how we interpreted the interviews. The adherence scores identified very high adherence behaviour by the ‘dominated’ group, whereas the ‘resistors’ had a low mean score with all scoring 7 or less apart from one participant (no.8, male, 55 years) who appeared to have recently improved his photoprotection following the scare of squamous cell carcinoma (Table 1). Overall the ‘integrated’ group had a moderate score, although half had a low score indicating that their routines were often not sufficiently rigorous.(Table 1).

Table 1: Specialist nurses assessment of photoprotection score [(a)] for three behavioural categories

Discussion

This is the first study to explore issues of adherence with photoprotection among people with XP and confirmed clinical perceptions of considerable variation in adherence. The main drivers influencing photoprotection behaviours broadly correspond with Horne and colleagues ‘Necessity-Concerns’ framework[24] developed to explain medicines use. However for photoprotection this involved balancing the perceived necessity of photoprotection to reduce UVR risks with concerns about the impacts of photoprotection on activities, feelings of stigma and emotional distress. For most younger ‘resistors’ their immediate psychosocial concerns and priorities outweighed their perceptions of risks and future impacts. They thus corresponded to what Sanderson et al [25] referred to in a study of rheumatoid arthritis as ‘struggling for normality at all costs’, possibly trading short-term social normality for normality in the longer term. There was some evidence that this emphasis on resisting photoprotection may have been influenced by earlier childhood experiences of negative responses and bullying.[26] In contrast, resistance among older people was mainly linked with questioning photoprotection as a personal priority.

For those ‘dominated’ by requirements for rigorous photoprotection their main driver was concern about UVR risks and future clinical impacts that outweighed the current

For peer review only - http://bmjopen.bmj.com/site/about/guidelines.xhtml

For peer review only

personal psychosocial impacts of photoprotection. This led to what Sanderson and colleagues referred to as a ‘disrupted normality’, although for XP this disruption occurred in social and psychological terms rather than through overwhelming symptoms.[27] The ‘integrated’ group in contrast with other groups rarely referred to particular motivators for photoprotection as by this stage their photoprotection had moved from a deliberative to an automatic and habitual process and was ‘*just part of life’* and therefore had a ‘reset normality.’

The experience and responses of patients with XP has parallels with the management of many chronic conditions including psoriasis, eczema, acne and other skin conditions for which internalized feelings of stigma are common and impact on quality of life in terms of work, social activities and relationships.[28] For people with XP their concerns however went beyond their facial appearance with the requirements of photoprotection forming a highly visible marker. The responses of younger ‘resistors’ were also similar to young adults with other chronic conditions including asthma, diabetes and epilepsy, who often lack the confidence and skills to reject stigma and have been described as engaging in ‘distancing’ and ‘denial’, thus forming a particularly hard to reach group in terms of behavioural change.[28-30]

The interviews focused on participants’ current mode of adjustment. However, a few people described transitions from ‘resisting’ to ‘dominated’ triggered by the experience of a basal cell carcinoma or melanoma. There was also evidence of a further shift towards ‘integration’ with increasing age, greater resilience and changing expectations. Many people were therefore leading a more normal life that integrated photoprotection, corresponding with the final stage of the self-management pathway in chronic illness.[31] However as Moss-Morris[32] noted there is need for a more adequate understanding and theory of adaptation to chronic illness. Similarly, Leventhal and colleagues[33] recommended that greater attention should be given to the dynamic processes involved in coping with chronic illness and key transitions, including transitions in relation to adherence.

Study limitations. The study of a rare disease involves a number of challenges.[34] A key challenge is associated with a necessarily limited sample that requires particular attention to ensuring confidentiality and anonymity. Study numbers assigned to

For peer review only - http://bmjopen.bmj.com/site/about/guidelines.xhtml

participants therefore differ from those identified by the clinic and only the gender and age of participants are reported. Studying a rare disease also leads to risks of considerable respondent burden. For example, it was not feasible to undertake further follow up interviews to explore emerging issues in greater depth or to undertake respondent validation as this patient sample was also participating in the other phase 1 studies and will be be involved in the subsequent trial. A further limitation is that clinic attenders with neurodegeneration, which occurs in around a quarter of people with XP,[9] were excluded as they are likely to experience different issues to people without neurodegeneration and thus require a separate study. The many requirements for rigorous photoprotection and individuals varying circumstances also identify a complex picture with triggers and processes of change between different behavioural response requiring detailed study with a longitudinal design.

Informing behavioural interventions This qualitative study complements the phase 1 survey and photoprotection activity data recorded in the N-of-1 study by providing greater depth and richness of data to explain participants behaviours and describe practices in greater detail. For example, why participants went out in the sun without full protection, what they thought about their social support, whether and what they disclosed to friends and how they responded to questions about their appearance.

These findings will inform the design of an individualized intervention. This includes identifying people *'dominated'* by photoprotection who already have a very high level of adherence and for whom the intervention would therefore not be appropriate. However, their accounts of emotional distress identified the potential risks associated with high levels of photoprotection and indicates that emotional well-being is an aspect that will need to be probed in the series of intervention sessions and responded to for individuals as appropriate.

The qualitative research also emphasised the importance of developing positive 'habits' which were common among the '*integrated'* group. This refers to a situation in which a stimulus (e.g. going outside) generates an impulse to act as a result of a learned stimulus response, with a positive cue-action link leading to protective behaviours.[36] Habit formation training could involve action planning and the use of environmental cues

For peer review only - http://bmjopen.bmj.com/site/about/guidelines.xhtml

and prompts (e.g. intervention text message) to instigate the specific photoprotection behaviour.

The study identified people 'who were *resistant*' to photoprotection as having few established routines and most likely to experience a constellation of barriers to photoprotection, while those categorized as *'integrated'* shared some of these barriers. This indicates that the intervention should be personalized to the barriers relevant to each person. Personalisation could involve completion of a short questionnaire to identify patterns of beliefs associated with '*resistant*' and '*integrated*' responses and discussion of facilitators and barriers in the initial session. Components to address specific needs and barriers might include strategies to enhance acceptance and willingness to photoprotect and to reduce felt stigma by managing appearance concerns.

Patients' direct accounts of their lived experiences elicited in the qualitative interviews could be presented as part of the written materials to support the one-to-one sessions. This is likely to assist individuals in overcoming personal barriers to photoprotection through understanding the experiences and views of others.

Conclusions

This study goes beyond the identification of themes to examine the complexity and patterning of responses to photoprotection, including the influence of patients' illness experience, priorities and circumstances of their lifeworld.[36] These data will inform the focus and design of individualized interventions to address motivational and volitional barriers to photoprotection for people with XP and may also be of value for other skin conditions that attract a stigma or require rigorous UVR protection.

References

1. Dunbar- Jacob j, Mortimer-Stephens MK. Treatment adherence in chronic disease. *J Clin Epidemiology* 2001; S4 (Supp 1): 557-60

2. Middleton KR, Anton SD, Perri MG. Long-term adherence to behavior change. *Am J Lifestyle Med* 2013; 7(6):395-404. Doi: 10.1177/155982761348867

For peer review only - http://bmjopen.bmj.com/site/about/guidelines.xhtml

3. Cutler R.L., Fernandez- Linos, F., Frommer, M, Benrimoj C, Garcia-Cardenas V. Economic impact of medication non-adherence by disease groups: a systematic review. *BMJ Open* 2018 Doi: 10.1136/bmj open-2017-016982

4. Vermieire E, Hearnshaw H, Van Royen P, Derekens J. Patient adherence to treatment: three decades of research. A comprehensive review. *Clinical Pharmacy Therapy* 2001; 26(5):331-342.

5. World Health Organisation, Adherence to long-term therapies: Evidence for action. *World Health Organisation* 2003: WHO/MNC/03,01

6. Cooper V, Clatworthy J. Unique patient challenges and support solutions in rare disease. *Atlantis Healthcare* 2014. https://www.google.co.uk/search?q=6.+Cooper+V%2C+Clatworthy+J.+Unique+patient+challenges+and+support+solutions+in+rare+disease.+Atlantis+Healthcare

7. Fassihi, H., Sethi, M., Fawcett, H. Wing, J., Chandler, N., Mohammed, S……..Lehmann, A.R. Deep phenotyping of 89 Xeroderma Pigmentosum patients reveals unexpected heterogeneity dependent on the precise molecular defect. *Proceedings of National Academy of Science USA*, 2016;113(9): E1236-45. doi: 10.1073/pnas.1519444113.

8. Kleijer WJ, Laugel V, Berneburg M, Nardo T, Fawcett H, Gratchev A, Jaspers NG, Sarasin A, Stefanini M, Lehmann AR.Incidence of DNA repair deficiency disorders in western Europe: Xeroderma pigmentosum, Cockayne syndrome and trichothiodystrophy. *DNA Repair* 2008; 7: 744–750.

9. Bradford, P.T., Goldstein, A.M., Tamura, D., Khan, S.G., Veda, T., Boyle, J…… Kraemer KH. Cancer and neurologic degeneration in xeroderma pigmentosum: long term follow-up characterises the role of DNA repair. *Journal of Medical Genetics* 2011; 48 (3):168-176.

10. Sethi, M., Lehmann, A.R., Fawcett, H., Stefanini, M., Jasprrs, N., Mullard, K., Turner, S……Fassihi, H. (2013). Patients with xeroderma pigmentosum complementation groups C, E and V do not have abnormal sunburn reactions. *British Journal of Dermatology* 2013; 169(6): 1279-86.

11. Tamura, D., DiGiovanna, J.J., Khan, S.G. et al. Living with xeroderma pigmentosum: comprehensive photoprotection for highly photosensitive patients, *Photodermatology, Photoimmunology & Photomedicine* 2014; 30(2-3): 146-152.

12. Antov D., Hollunder, M., Schliemann, S., Elsner, P. Ultraviolet exposure and protection behavior in the general population: A structured interview survey. *Dermatology* 2016; 232:11-16.

For peer review only - http://bmjopen.bmj.com/site/about/guidelines.xhtml

13. Diao DY, Lee TK. Sun protective behaviours in populations at high risk of skin cancer. *Psychol Res Behav Manag* 2014; 7: 9–18.

14. Skiveren J, Mortensen EL, Haedersfdal M. Sun protective behavior in renal transplant recipients. A qualitative study based on individual interviews and the Health Belief Model. *J Dermatol Treat* 2010; 21:331-336

15. Fassihi H. Personal communication.

16. Team protocol paper

17. Bartholomew, K., Parcel, G.S., & Kok ,G. Intervention mapping: A process for developing theory – and evidence-based health education programs. *Health Education & Behaviour 1998;* 25(5): 545-563.

18. J. Ritchie & J. Lewis (Eds.), *Qualitative research practice : a guide for social science students and researchers* 2003: 219-262. London: Sage.

19. O'Brien, B.C., Harris, IB, Beckman T.J., Reid, D.A., Cook, D.A. *Standards for reporting qualitative research: a synthesis of recommendations*. Acad Med.2014;89(9):1245-1251.

20. Richie, J., Spencer, L., & O'Connor, W. Carrying out qualitative analysis. In J. Ritchie & J. Lewis (Eds.), *Qualitative research practice : a guide for social science students and researchers 2003*, 219-262. London: Sage.

21. Lincoln, Y.S., Guba, E.G. *Naturalistic Inquiry* 1995. Newbury Park CA, Sage Publications.

22. Henshaw T, Turner S. Audit of ultraviolet radiation practices by patients with xeroderma pigmentosum. *Dermatological Nursing* 2016; 5 (3): 65.

23. Scambler, G. Review article: Health-related stigma. *Sociology of Health & Illness 2009;* 31(3): 441-455.doi: 10.1111/j.1467-9566.2009.01161.x

24. Horne, R., Chapman, S.C.E., Parham, R., Freemantle, N., Forbes, A. & Cooper, V. Understanding patients' adherence-related beliefs about medicines prescribed for long-term conditions: A meta-analytic review of the Necessity-Concerns framework. *Plos One* 2013; 8(12) :e80633.

25. Sanderson, T., Calnan, M., Morris, M., Richards, P., & Hewitt, S. Shifting normalities: interactions of changing conceptions of a normal life and the normalization of symptoms in rheumatoid arthritis. *Sociology of Health & Illness* 2011; 53(4):618-623.

26. Team paper

For peer review only - http://bmjopen.bmj.com/site/about/guidelines.xhtml

27. Foggin E, Cuddy L, Young H. Psychosocial morbidity in skin disease. *British Journal of Hospital Medicine* 2017; 78(6): C82-C86. doi: 10.12968.hmed.2017.78.6.C82.

28. Bulow, P., Palmer, S., Pai, A., Goodenough, B., Luckett T., King, M. Review of adherence-related issues in adolescents and young adults with cancer. J*ournal of Clinical Oncology* 2010; 28(32): 4800-4806.

29. Johnson, H.M., Warner, R.C., LaMantia, J.N., Bowers, B.J. "I have to live my life like I'm old. "Young adults' perspectives on managing hypertension: a multi-center qualitative study. *BMC Family Practice 2016.* doi 10.1.1186/s12875-016-0428-9

30. Oris L, Rassart J, Prikken S, Verschueren M, Goubert L, Moons P, Berg CA, Weets I and Luyckx K. Ilness identity in adolescents and emerging adults with type 1 diabetes: Introducing the illness identity questionnaire Diabetes Care 2016 May; 39(5): 757-763. doi.org/10.2337/dc15-2559

31. Audulv, A., Asplund, K. & Norbergh K-G. The integration of chronic illness self-management. *Qualitative Health Research* 2012; 22(3): 332-345. doi:10.1177(1049732311430497

32. Moss-Morris R. Adjusting to chronic illness: Time for a unified theory. British *Journal of Health Psychology* 2013; 13, 8(4): 681-686. doi.org/10.1111/bjhp.12072

33. Leventhal, H., Phillips L. A. & Burns E. The Common-Sense Model of Self-Regulation (CSM): a dynamic framework for understanding illness self-management. *Journal of Behavioural Medicine* , 2016,39, 933-946. doi 10.1007/s10865-016-9782-8.

34. Sainsbury, K., Walburn, J., Araujo-Soares, V., Weinman. J. Challenges and proposed solutions for formative research to inform systematic intervention development in rare and unstudied conditions: The case example of Xeroderma Pigmentosum. *British Journal of Health Psycholgy* 2017; 23: 229-237.

35. Gardner B, Lally P, Wardle J. Making health habitual: the psychology of 'habit – formation' and general practice. Br J Gen Pract 2012, 62(605): 664-666.

36. Mishler, E. 'Viewpoint: critical perspectives on the biomedical model'. In Mishler, E., Amara Singham, L., Hauser, S., Liem, S., Osherson, R. and Waxler, N. (eds) *Social Contexts of Health, Illness and Patient Care*. Cambridge: Cambridge University Press, 1981.

Acknowledgements : We are most grateful Lesley Foster who was instrumental in recruitment and facilitating the interviews. We also thank the clinical specialist XP team

For peer review only - http://bmjopen.bmj.com/site/about/guidelines.xhtml

at St Thomas' Hospital for helpful discussions and specialist nurses Tanya Henshaw & Sally Turner for adherence data. We benefitted from input by the PPI panel (Cathy Coleman, Ben Fowler, Ros Tobin, Sandra Webb) and are most grateful to the study participants for the detailed accounts of their experiences and photoprotection practices.

Contributors: MM designed the study with input from RS &JW and was involved in supervising the study, analyzing data and writing the paper. RA & J Wal conducted interviews, checked transcripts and contributed to development of project materials and interpretation of the findings. MM and JWal conducted the clinical discussion groups and JWal held the PPI discussion. All authors made critical revisions to the paper.

Funding: This article presents independent research funded by the National Institute for Health Research (NIHR) under its Programme Grants for Applied Research scheme (RP-PG-1212-20009). The views expressed are those of the authors and not necessarily those of the NHS, the NIHR or the Department of Health. NIHR had no involvement in study design, research process or manuscript preparation.

Conflicts of interest: None declared

Ethics approval: London-Camden & Kings Cross Research Ethics Committee (REC number 15/LO/1395)

Data sharing statement: No additional data are available

For peer review only

For peer review only

For peer review only - http://bmjopen.bmj.com/site/about/guidelines.xhtml

Table 1. Specialist nurses assessment of photoprotection score [(a)] for three behavioural categories

Behavioural categories	Range	Mean protection practice score (SD)	Protection score ratings [(a)]
Dominated	16-20	18.25 (2.06)	High
Resistant	3-14	6.18 (3.25)	Low
Integrating	7-15 [(b)]	9.90 (3.00)	Moderate

(a) Based on Ultraviolet Radiation Protection Measure applied by specialist nurses (Henshaw & Turner, 2016). Scores out of 20 based on 13 items covering 5 areas:
1.Window film 2. Sunscreen 3. Face visor or Glasses/UVR eye protection/Face buff/Hat 4. Hand protection 5. Arms/legs covered.
High score = 15-20, Moderate score = 9-14, Low score = 0-8

(b) The score does not take account of UVR protection with heavy curtains or blinds, with only dermaguard window film at home receiving a score (100% day protected = 6; 50% day=3). This reduced the score for people who spent most of their time indoors with curtains or blinds pulled.

For peer review only

Figure 1.

Behavioural responses to photoprotection among people with XP

Dominated by demands of photoprotection: Major motivator for rigorous photoprotection was high fear of UVR related risks. Although concerned about the impacts of photoprotection on activities and experienced emotional distress, these did not lead to reduced photoprotection.

Resistant to photoprotection: Major motivator was the desire to live a normal life which conflicted with rigorous photoprotection.
For a group of younger adults their desire to distance themselves from a stigmatized identity and be accepted as normal by their peer group outweighed concerns about the clinical risks of UVR. This led to concealing their condition by not undertaking rigorous photoprotection and often not disclosing their condition to others.
For older adults the requirements for photoprotection had no significant impact on self-identity or disclosure. However there were gaps in their practices, especially going out for short periods without sunscreen, reflecting a lack of motivation due particularly to their questioning of the personal risks of UVR, its perceived efficacy and the priority given to other things in life.

Integrated photoprotection: UVR protection integrated into normal life and not perceived as disruptive to activities or an emotional burden.
Some achieved this by largely staying indoors which they were used to from childhood and was now their preferred life style. Others lived a more usual life with UVR protection assisted by the co-operation of friends/family and among older people by the changing values, lifestyles and expectations of older age. Many participants although confident in their photoprotection also acknowledged some scope for improving routines.

For peer review only - http://bmjopen.bmj.com/site/about/guidelines.xhtml

For peer review only

Supplementary Table 1: Reporting checklist for qualitative study.

		Reporting Item	Page Number
	#1	Concise description of the nature and topic of the study identifying the study as qualitative or indicating the approach (e.g. ethnography, grounded theory) or data collection methods (e.g. interview, focus group) is recommended	p.2 Abstract
	#2	Summary of the key elements of the study using the abstract format of the intended publication; typically includes background, purpose, methods, results and conclusions	p.2 Abstract
Problem formulation	#3	Description and signifcance of the problem / phenomenon studied: review of relevant theory and empirical work; problem statement	pp.4-5
Purpose or research question	#4	Purpose of the study and specific objectives or questions	p.5
Qualitative approach and research paradigm	#5	Qualitative approach (e.g. ethnography, grounded theory, case study, phenomenolgy, narrative research) and guiding theory if appropriate; identifying the research paradigm (e.g. postpositivist, constructivist / interpretivist) is also recommended; rationale. The rationale should briefly discuss the justification for choosing that theory, approach, method or technique rather than other options available; the assumptions and limitations implicit in those choices and how those choices influence study conclusions and transferability. As appropriate the rationale for several items might be discussed together.	p.5, p7
Researcher characteristics and reflexivity	#6	Researchers' characteristics that may influence the research, including personal attributes, qualifications / experience, relationship with participants, assumptions and / or presuppositions; potential or actual interaction between researchers' characteristics and the research questions, approach, methods, results and / or transferability	p.6
Context	#7	Setting / site and salient contextual factors; rationale	p.7
Sampling strategy	#8	How and why research participants,	p.7,

For peer review only

		documents, or events were selected; criteria for deciding when no further sampling was necessary (e.g. sampling saturation); rationale	
Ethical issues pertaining to human subjects	#9	Documentation of approval by an appropriate ethics review board and participant consent, or explanation for lack thereof; other confidentiality and data security issues	p.20
Data collection methods	#10	Types of data collected; details of data collection procedures including (as appropriate) start and stop dates of data collection and analysis, iterative process, triangulation of sources / methods, and modification of procedures in response to evolving study findings; rationale	p.6-7
Data collection instruments and technologies	#11	Description of instruments (e.g. interview guides, questionnaires) and devices (e.g. audio recorders) used for data collection; if / how the instruments(s) changed over the course of the study	p.28-29
Units of study	#12	Number and relevant characteristics of participants, documents, or events included in the study; level of participation (could be reported in results)	p.8
Data processing	#13	Methods for processing data prior to and during analysis, including transcription, data entry, data management and security, verification of data integrity, data coding, and anonymisation / deidentification of excerpts	p.7 &
Data analysis	#14	Process by which inferences, themes, etc. were identified and developed, including the researchers involved in data analysis; usually references a specific paradigm or approach; rationale	p. 7
Techniques to enhance trustworthiness	#15	Techniques to enhance trustworthiness and credibility of data analysis (e.g. member checking, audit trail, triangulation); rationale	p.7, p.3
Syntheses and interpretation	#16	Main findings (e.g. interpretations, inferences, and themes); might include development of a theory or model, or integration with prior research or theory	pp.8-14
Links to empirical data	#17	Evidence (e.g. quotes, field notes, text excerpts, photographs) to substantiate analytic findings	pp.8-14

For peer review only

For peer review only - http://bmjopen.bmj.com/site/about/guidelines.xhtml

Intergration with prior work, implications, transferability and contribution(s) to the field	#18	Short summary of main findings; explanation of how findings and conclusions connect to, support, elaborate on, or challenge conclusions of earlier scholarship; discussion of scope of application / generalizability; identification of unique contributions(s) to scholarship in a discipline or field	pp.16-17
Limitations	#19	Trustworthiness and limitations of findings	p.15
Conflicts of interest	#20	Potential sources of influence of perceived influence on study conduct and conclusions; how these were managed	n/a
Funding	#21	Sources of funding and other support; role of funders in data collection, interpretation and reporting	p.19

The SRQR checklist is distributed with permission of Wolters Kluwer © 2014 by the Association of American Medical Colleges. This checklist can be completed online using https://www.goodreports.org/, a tool made by the EQUATOR Network in collaboration with Penelope.ai

Supplementary Table 2: Themes and examples of open questions from topic guide

---

Introduction

Explanation that this is the first time research has been done in XP from the patients perspective and we want to learn more about the day to day experiences and challenges of living with XP from your personal point of view. We are having this conversation to find out what it is like living with XP. We are interested in your views and understanding XP from your perspective. There are no right or wrong answers.

The interview is confidential and when we write up the study any quotations that we use will be anonymous and will not be linked to your name. The staff at the XP clinic will not hear your interview and will not know what you have said.

Taking part in the interview is voluntary - you can stop at any time. If you want to stop just let me know. If you want to take a break, just let me know. You can take your time to think about what you want to say – there is no rush.

In the information leaflet we explained that we would like to record the conversation. Is this still OK with you? This is to help us with the analysis.

Topics and examples of questions

1. Participants personal story of their XP
When were you first aware of having a skin problem? How was it diagnosed? What was your understanding of XP at that time?
What do you think are the risks of XP for you? How much does this bother you?
For how long have you been attending the specialist clinic at XX? Has this been helpful?

2. General views of photoprotection
How important is photoprotection for you? Do you think it is important in all seasons including winter and on cloudy days?
What are any particular downsides of photoprotection for you?

3. Photoprotection practices
How important is sunscreen for you? How regularly do you use sunscreen?
Do you go out without using? Are there any problems with it?

Do you make special efforts to wear clothing to cover up including gloves, long sleeves, long trousers and hats or hoodie? Glasses? Is this protection easy or are there problems with having to cover up like this?

Do you wear a visor? Is it comfortable? How important to use? If not wear currently - have you ever worn one? Why did you stop?

Do you avoid going out when sunny? How does this restrict your life?
Do you do any UV protection when inside?
Have there been any changes in your photoprotection over time?

4. **Psychosocial burden of XP/photoprotection**
What are the main ways having XP affects your activities, such as work/school/leisure activities?

What are your biggest worries about having XP?
Does having XP affect you emotionally?

5. Support by family and friends
Do other people understand/support you? In what ways? How important/helpful is this?

Do you worry about what others think about you?
Do you think others respond to you differently because of your XP?
Have you had any experiences that have made you feel this way? How do you cope with this?

For peer review only - http://bmjopen.bmj.com/site/about/guidelines.xhtml

For peer review only

For peer review only - http://bmjopen.bmj.com/site/about/guidelines.xhtml

BMJ Open

The influence of perceived medical risks and psychosocial concerns on photoprotection behaviours among adults with Xeroderma Pigmentosum: A qualitative interview study in the UK

Journal:	*BMJ Open*
Manuscript ID	bmjopen-2018-024445.R1
Article Type:	Research
Date Submitted by the Author:	05-Sep-2018

Complete List of Authors:	Morgan, Myfanwy; King's College London, School of Cancer and Pharmaceutical Sciences<br>Anderson, Rebecca; King's College London, School of Cancer and Pharmaceutical Sciences<br>Walburn, Jessica; King's College London, School of Cancer and Pharmaceutical Sciences<br>Weinman, John; Kings College London, School of Cancer and Pharmaceutical Sciences & Institute of Psychiatry<br>Sarkany, Robert ; Guy's and St Thomas' NHS Foundation Trust, St John's Institute of Dermatology

**Primary Subject Heading**:	Dermatology
Secondary Subject Heading:	Qualitative research, Health services research
Keywords:	Adult dermatology < DERMATOLOGY, Adherence, Health Behaviours, Photoprotection, QUALITATIVE RESEARCH

SCHOLARONE™
Manuscripts

The influence of perceived medical risks and psychosocial concerns on photoprotection behaviours among adults with Xeroderma Pigmentosum: A qualitative interview study in the UK

Myfanwy Morgan,[1] Rebecca Anderson,[1] Jessica Walburn,[1] John Weinman,[1] Robert Sarkany. [2]

[1] School of Cancer and Pharmaceutical Sciences, King's College London, UK

[2] National Xeroderma Pigmentosum Service, Guy's and St.Thomas' NHS Foundation Trust, London, UK

Corresponding author: Myfanwy Morgan

King's College London,
School of Cancer and Pharmaceutical Sciences,
152 Stamford Street, London SE1 9NH

Email: myfanwy.morgan@kcl.ac.uk

Key words: adherence, dermatology, photoprotection, appearance, qualitative.

For peer review only

Abstract

Background: A high level of photoprotection is required by people with Xeroderma Pigmentosum (XP), a rare skin disease, to reduce skin cancer and other risks. However poor photoprotection is thought to be widespread.

Purpose: This study examines the influences on photoprotection behaviours in adults with XP.

Design: Inductive qualitative study with semi-structured interviews. Analysis employed a Framework approach.

Setting: National sample recruited through a specialist XP centre in London.

Methods: Semi-structured interviews at patients' home. All transcripts were coded and themes charted for each participant. Comparisons within and across cases identified common themes and differing motivations and approaches to photoprotection. Credibility of interpretations assessed through patient/carer input and clinic adherence scores.

Participants: 25 adults (17 males, 8 females) aged 16-63 years with diagnosed XP attending a specialist centre. Eighteen lived outside London.

Results: Awareness of risks of UVR and photoprotection was high. However, photoprotection behaviours varied according to perceived necessity and concerns. Three behavioural responses were identified: 1) *'dominated'* by planning and routines to achieve a high level of photoprotection with significant activity restrictions and psychosocial impacts; 2) '*resistant'* to photoprotection with priority given to avoiding an illness identity and enjoying a normal life; 3) photoprotection *'integrated*' with individual's life with little psychosocial impact. These responses were influenced by illness, personal and contextual factors including age, life stage and social support. Only the 'integrated' group achieved an equilibrium between perceived 'necessity' and 'concerns'.

Conclusions: The personal balance between perceived risks of UVR and social/ psychological 'concerns' led to differing behavioural responses and contributes to an understanding of adaptation and normalization in chronic illness. The study will also inform a series of individualized behavioural interventions to reduce measured UVR exposure among people with XP that are potentially applicable to other conditions with high risks of skin cancer.

296 words

For peer review only

Article Summary

Strengths and limitations of the study

Qualitative home based semi-structured interviews elicited detailed accounts of individuals' perceptions and photoprotection behaviour.

The analysis went beyond identification of individual themes to identify three multi-dimensional categories of response to photoprotection, with their credibility enhanced through discussion with the patient/carer (PPI) group and data on prior specialist nurse ratings of adherence.

The research was conducted with patients in the National Health Service who attended a national specialist centre for XP which may have influenced their knowledge and attitudes to XP.

It was not feasible to conduct follow-up interviews to examine the influences on transitions between different modes of response given the significant demands of the overall research programme on a small sample of people with this rare disease.

.

For peer review only

The influence of perceived medical risks and psychosocial concerns on photoprotection behaviours among adults with Xeroderma Pigmentosum: a qualitative interview study in the UK

Introduction

Globally the prevalence of chronic disease is increasing but adherence with recommended therapies remains persistently low; on average 50% of all
patients with long-term therapy for chronic disease in developed countries are non-adherent with medication[1] and 50-80% for behavioural changes.[2] Both forms of non-adherence increase risks of morbidity and mortality with implications for demands on health services and costs of care.[3]

Research conducted to explain high levels of non-adherence with medical advice has identified a range of illness related, psychosocial and social structural influences.[4,5] However, very few studies have examined the determinants of non-adherence among people with a rare disease, defined by the European Union as conditions that affect fewer than 5 in 10,000 people in the general population.[6] Rare diseases in totality are however not so rare, with over 6000 rare diseases identified. It is also estimated that 7% of the population will be affected by a rare disease at some point in their lives.[6]

This study focuses on adherence with recommended lifestyle changes among patients with Xeroderma Pigmentosum (XP), a rare disease caused by mutations in any of eight genes, resulting in defective repair of ultraviolet radiation (UVR) induced DNA damage.[7] The incidence is 2.3 per million live births in Western Europe.[8] Patients may develop skin cancers from childhood onwards, with an estimated 2,000 and 10,000 fold increased risk of melanoma and non-melanoma skin cancers respectively, together with increased risks of ocular problems and neurodegeneration.[9] About half the patients were 'burners' who suffer abnormal and severe sunburn reactions and blistering as measured by an XP sunburn score and are at the highest risk of neurological degeneration. 'Non-burners' have an entirely normal sunburn response but have the highest risks of pigmentation changes, skin cancers at an early age and ocular problems.[10]

There is currently no curative treatment for XP. Improving life expectancy and reducing risks of skin cancers and eye disease therefore depends on rigorous long-term UVR protection. This involves regular use of sunscreen (SPF 50+), covering arms and legs with layers of clothing and wearing gloves, a UVR protective visor or other head/face covering, UVR

For peer review only - http://bmjopen.bmj.com/site/about/guidelines.xhtml

protective glasses and attaching UVR protective film to windows.[11]

Research to date has shown that sun protective knowledge is high among general populations[12] and among high risk groups [13,14] but has not adequately explained low adherence. Furthermore, no research has focused specifically on people with XP, although expert clinical opinion suggests these patients may vary widely in the degree to which they photoprotect.[15]

The current study forms one component of a five year NIHR funded programme of research that aims to develop and evaluate interventions to increase adherence with photoprotection and thus reduce measured UVR exposure among people with XP.[16] The programme is underpinned by a systematic theoretically based framework to guide behavior change interventions.[17] The first mixed methods phase therefore aimed to achieve a comprehensive understanding of photoprotection behaviour to inform the development of individualized photoprotection interventions.[16] The quantitative studies comprised a cross-sectional survey to assess potential clinical, socio-demographic and behavioural correlates of photoprotection behavior and an N-of-1 study using daily diaries to record photoprotection activities and ratings of potential psychosocial predictors. These were complemented by this more in-depth qualitative study to explain photoprotection behaviour.

Aims

To elicit individuals' personal accounts of their photoprotection behaviour and identify the range of factors that influence their behaviour.

Methods

Design: Inductive qualitative study based on semi-structured interviews that were analysed following a Framework approach.[18] The Standards for the Reporting of Qualitative Research (SRQR) guidelines were followed.[19] (Additional Table)

Patient and public involvement: Six members of the Patient and Public Involvement (PPI) group were recruited. However, two parents dropped out as their child was having severe health problems. The remaining four PPI members (1 patient, 2 parents, 1 teacher) participated in two discussion sessions to consider the qualitative findings. One parent was a co-investigator and as part of the research team attended meetings throughout the research from the initial development of the application.

For peer review only

All study participants were sent a brief newsletter that provided study feedback. They were also invited to a thank you event that included informal discussion of the results. Further dissemination will occur following the trial phase of the research.

Recruitment: The study sample were recruited from the national specialist XP service in London that is part of the National Health Service with universal access based on medical need. Eligible adults were identified from the register of clinic attenders and satisfied the following criteria: aged 16 years and over, confirmed diagnosis of XP (reduced unscheduled DNA repair in fibroblast DNA repair assay), no neurodegeneration, adequate English to participate in an interview, and had not opted out of research.

A research nurse who was employed by the research programme and had no clinical contact with patients organized study recruitment. She sent study information to the 38 eligible patients and two weeks later followed up with a phone call to enquire whether they were interested in participating. Seven could not be contacted and six declined due to time or not wishing to talk about XP. These six patients comprised three men and three women with a spread of ages. Their clinic notes also identified varying photoprotection behaviours thus identifying their similarity to the interviewed sample.

Data collection: Twenty-five one-to-one interviews interviews were conducted. They were held in a private room in the patient's home with the exception of one phone interview for logistical reasons. Interviews were carried out between early February and June 2016 by a Research Fellow (JWal) and Research Assistant (RA) who are both trained in health psychology and experienced interviewers but had not previously studied XP. Participants were aware that they were non-clinical researchers employed at King's College London to conduct the research. An interviewer (female) visited the patient's home with the research nurse who had made the initial contact with participants to provide continuity. She introduced the interviewer and obtained informed consent but was not present for the interview and when required looked after participants' children.

Ethics approval was granted by London-Camden & Kings Cross Research Ethics Committee (REC number 15/LO/1395)

Interviews took the form of a guided conversation structured by a topic guide (Supplementary Table 1). They began with a brief reminder of the aims of the study and then focused on aspects of the patients' personal story to identify individuals' early experience of XP. This was followed by eliciting their current views and experiences of photoprotection, the

For peer review only

psychosocial impacts of photoprotection and support by family and friends. Topics were discussed in any order according to the flow of the conversation and responses probed and clarified as necessary. Interviews were audio recorded with permission and mainly took 30-60 minutes. Participants were generally willing to provide detailed accounts of their photoprotection behaviour. This is likely to have been facilitated by the home environment and their positive views of both the clinic and the aims of the research. However reflective notes made following the interview identified a few participants becoming upset in thinking about XP. Some also preferred not to discuss earlier stages and only to focus on the present. However, they were all happy to continue the interview.

Data saturation was achieved with the 25 interviews with no new beliefs or explanations identified in the final interviews.

Analysis: This occurred alongside data collection and was based on a Framework approach.[18] This goes beyond a thematic analysis and involves methods that are geared towards producing practice-oriented findings and was therefore appropriate for a study aiming to inform behavioural change interventions.[20]

Tapes were transcribed verbatim, checked for accuracy and corrected transcripts entered into QSR NVivo11 which is a qualitative data analysis package designed for working with text or multi-media information where in-depth analysis is required (http://www.qsrinternational.com/products_nvivo.aspx).Transcripts and fieldnotes were initially read to identify key themes. Coding was then undertaken with the initial coding framework adapted as new items arose. One author (RA) had a major role in coding (i.e.' indexing') with two other authors (JWal, MM) repeating and reviewing the coding which was discussed to achieve consensus. Analysis involved comparisons within and across cases assisted by 'charting' which involves summarizing data by thematic content for each case to identify relationships between codes.[20] These charts were further examined through a process of 'mapping and interpretation' leading to emerging ideas and relationships. Preliminary behavioural categories were tested through further analysis of transcripts to identify the fit for all cases with modifications introduced as required. This iterative process finally led to a 3-fold categorization based on perceptions of risks of UVR and the psychosocial impacts of photoprotection.

Credibility and triangulation.

The credibility of emergent categories in terms of their congruence with reality forms a key aspect of validity in qualitative research.[21] In this case credibility was informed by the patient

For peer review only

and public involvement (PPI) group who participated in two sessions to discuss the qualitative findings and in two clinical discussion groups held with a total of eleven clinical specialists who undertake the patients' annual check to identify the congruence of our identified patient categories with their clinical experience. The level of adherence with photoprotection of our three patient categories was also examined based on prior clinic reviews by specialist nurses using a 13-item measure.[22]

Results

Characteristics of participants: Twenty-five adults were interviewed. Participants were aged 16-63 years and 18 lived outside London. Seventeen were male and eight female reflecting the adult clinic caseload (26 males and 17 females). Eight were European (mainly white British), eight of Pakistani ethnicity and seven mainly of Middle Eastern, Indian or Bangladeshi background reflecting the relatively high prevalence of XP in Pakistan and the Middle East.[7]

Fifteen participants were defined as 'non-burners' (i.e. normal sunburn response) and ten as 'burners' (i.e. abnormal sunburn response to minimal sun exposure).[10] Those categorized as 'burners' had all begun some form of photoprotection in childhood following recognition that they burned easily, whereas half the 'non-burners' had begun photoprotection after 10 years of age. Self-reported clinical diagnosis occurred more than 5 years ago for all but five participants and all currently attended the specialist clinic annually. The 'burners' had experienced reddening and blistering, pigmentation changes particularly freckles and benign lesions. The 'non-burners' had often experienced some pigmentation changes and nearly all had experienced removal of one or more cancerous lesions.

Knowledge of risks and photoprotection. Participants were all aware that XP is a long-term condition with risks of skin damage and lesions. They were also aware of requirements for photoprotection and explained that their knowledge had increased over time through greater experience of the condition and attending the specialist clinic. For example, some participants initially thought only the face is affected by UV exposure and were therefore surprised to require lesions removed from their neck, arms or legs, As participant 28 (female, age 28) explained:

''….so my neck, so then I'll wear a hat and I'll wear sun cream more often, and I'll wear my sunglasses. Then there's bits on your arms, so I'll start wearing long sleeve t-shirts. And then this happened on my leg…..but now I'm like no, I'm going to buy leggings and I'm going to wear leggings with long sleeve tops.'

For peer review only

All participants engaged in some photoprotection but when asked why they did not undertake particular aspects often found this difficult to answer. However, further discussion identified a range of positive and negative factors that influenced how they interpreted and balanced medical and psychosocial priorities. This led to identifying three main types of response to photoprotection: '*dominated*' by photoprotection, '*resistant'* to photoprotection and *integrated*' photoprotection (Box 1). See Supplementary Table 2 for participants' age/sex and behavioural response category.

Insert Box 1: Behavioural responses to photoprotection among people with XP

Responses to photoprotection

1. Dominated by demands of photoprotection. This group comprised four men (one 'burner' and 3 'non-burners') aged between 21 and 28 years who all described a very high level of photoprotection. Their major motivation for undertaking rigorous photoprotection was their perception of the harmful effects of UVR. The three non-burners feared cancerous lesions and worried about a reduced length of life, whereas the major worry for the one 'burner' (Participant 15 age 22) was having very painful and unpleasant sunburn reactions. However, for all participants an immediate concern was the effects of UVR on their appearance due to freckling and other pigmentation changes including facial scars following surgery. For example, Participant 14 a non-burner (male, age 21) explained, '*If you take it purely objectively, I don't think I'm as unfortunate as some skin patients are (effects on appearance). But you still worry about it. Yeah, that's a main worry.'*

People 'dominated' by photoprotection had begun sun protection in childhood but described adjusting better to it as they got older and often did not want to think back to earlier stages, describing this as '*too long ago.'* However, participant 07 (male, age 27) explained that at school and college he never used his sunscreen '*because I wanted to feel normal, that's why...I didn't want any questions, or any remarks from friends or people that I didn't know, and I did not want to look abnormal to anyone else.'* This changed when he had his first surgery to remove a basal cell carcinoma which he described as a '*reality check'* and '*wake-up call' as 'I was putting myself in harms' way for no reason…just destroying my own skin.*' From that time he gave considerable emphasis to the risks of UVR and the need for rigorous photoprotection.

All four respondents described having UVR protective film on the windows of their home and car and used a UVR meter to assess risks and identify appropriate environments and locations. They also described well-established behavioural routines for photoprotection. This involved applying sunscreen whatever the season and wearing two or three layers of protective clothing, gloves and a hat or cap with a hoodie or a visor when outside during the day. A visor provides full cover of the face and head and is usually only worn by children but was worn by three people categorized as 'dominated'. They described a visor as uncomfortable to wear, bulky to carry around and attracting stares and comments. However, users regarded this as necessary to achieve sufficient protection and regarded wearing a visor as part of '*going out properly protected*.' The one participant in the 'dominated' group who did not wear a visor but otherwise engaged in rigorous photoprotection described a visor as 'suffocating' and as having negative psychosocial impacts:

'..totally make me feel abnormal. Just wearing a hat, cap and glasses all *the time, I've gotten people questioning like mad, so imagine wearing a visor.'* (Participant 7, male, 27yrs)

Participants 'dominated' by photoprotection explained how their practices had shifted from a deliberative action into a routine or 'habit' which they now *'do without thinking, it becomes part of your daily routine*.' As one participant observed,

'I think I'm motivated by keeping myself safe and I don't want to develop any skin cancers, which is a problem for my condition. But really, rather than motivation it's something I do just because it's a deeply ingrained in me and because I've been doing it forever.' (Paricipant 13, male, age 28)

This high level of photoprotection carried considerable personal costs. These included restrictions on choice of job with risks of exposure to UVR forming an important consideration:

'It's affected the kind of jobs that I can look for. I only choose something that's inside in a safe environment and I really can't go for anything else.'. He now works as a shop assistant in a large supermarket (Participant 14, male, age 21). Others rather than doing their job of choice were employed in safe environments as a barber, in the family takeaway or as a health scientist.

Participants also described missing out on social events because they did not want to wear UVR protection or did not regard it safe to go outside even with full protection. For example, participant 13 (male, age 28) explained,

'I make excuses about not going out with friends and prefer to be indoors between 11 and 4 when the sun is at its highest.'

For peer review only

If going out during the daytime was unavoidable they undertook considerable preparation. As participant 22 (male, age 26) explained, '*the only times I go out during the day is if I'm going from a car to a building or building to car, or if it's completely unavoidable*….' This decision in turn cues his usual routine that involves '*suncream, visor, everything*.' He described this as taking him *'……..about half an hour just to get ready because I've got to apply my sun cream, then I've got to be sure I haven't missed any areas. I also apply lip balm which is UV protected. Then once I've applied my sunscreen I apply my camouflage (to hide scars). I apply two camouflages.'* He also explained, '*If I've got to be somewhere for 5pm I'll have to leave at 4.30pm which means I've got to cover up between (workplace) and the tube station. So, I need the visor that day.*'

This group referred to the psychological costs of a high level of photoprotection in terms of being viewed as different or not 'normal'. They described no longer being upset by stares and questions about their clothing and if people continued to ask questions or make comments they now felt more confident and able to '*put them in their* place.' However, they still sometimes avoided situations that might lead to '*snide remarks'*, such as taking a taxi rather than a bus when school children were travelling.

Despite considerable support from family and friends this group described feeling emotionally distressed about the demands of photoprotection and restrictions on their activities. For two participants the impacts were quite severe, for example participant 7 (male, age 27) explained, '*whatever I do XP is on my mind first'* and described XP as '*affecting me emotionally quite a bit as well.,,,,,It's just you get obsessed and get that depressing feeling. You can't do much in life.'* Similarly, participant 22 (male, age 26) described '*feeling down'* about having XP and its effects on his life and got '*bouts of depression*' about three or four times a year. However, these various adverse effects did not disrupt their high level of photoprotection given their fears of the potential clinical risks.

2. Resistant to photoprotection. This group comprised 11 participants, aged 20-55 years, with five 'burners' and six 'non-burners'. All accepted their diagnosis of XP and were aware of the risks of UVR and photoprotection practices. They therefore had the capability to photoprotect but acknowledged a lack of motivation with other priorities.

We identified five people (2 men and 3 women, aged 21-37 years) as 'strong resistors' as they described very limited photoprotection that largely reflected their desire to distance themselves from XP and be accepted as normal by others. For example, participant 24

(female, age 28) had experienced several surgeries to remove skin cancers and benign lesions and was particularly concerned about surgery to her face. Nevertheless, she described 'strong resistance' to photoprotection and often joined her friends for activities in the sun, did not consistently reapply sunscreen and sometimes went out wearing shorts or summer tops, because as she explained:

'*I just want to be seen like everybody else'* and "*I think you can just let these things (photoprotection) become you and I don't want to be about XP. XP is a tiny part of me.'* She therefore sometimes thinks, '…*do you know what, I'm just going to get on with my life.'* She excused her behavior at interview by saying that when she was out she 'shade hopped' and also gained reassurance from knowing that the clinic would identify and manage any problems.

Concerns about feeling different and viewed by others as possessing an unacceptable difference was common in this group although their particular circumstances differed. For example, an Asian participant (no. 6, male, age 20 years) described freckles as highly unusual and abnormal on a person with Asian skin and reported that comments and questions by the Asian community made him feel '*like an outsider'* which corresponds with the notion of 'felt stigma' or feelings of shame associated with an unacceptable difference. [23] This response contributed to his stress and loneliness and to a reluctance to increase the obviousness of his condition through rigorous photoprotection.

This sub-group group of 'strong' resistors therefore described tensions between their desire to be seen and participate as normal and worries about the effects of UVR without sufficient protection. For example, participant 18 (female, age 27) worries about cancer but often feels '*a bit left out'* as she can't go outside at lunch with colleagues. She also acknowledged that although she sometimes wears long sleeves in summer she does not usually wear a hat or reapply sunscreen which she regarded as greasy and staining. She explained that '*some days I don't bother with cream or covering up…..Sometimes I think screw it, just go out. Then I think, oh, sugar,….But there's consequences, just like oh my god.'* Similarly participant 12 (male, age 26) explained that he worries about life expectancy and appreciates the advice and support given by the clinic but still goes out and socializes in the summer'…*just to make myself feel better really, That's about it.*'

Concerns about being perceived and responded to in terms of XP led to concealing the condition by reduced photoprotection, the use of a tinted foundation with a lower SPF and reluctance to disclose their condition to others. Reminders to photoprotect by family members were also often described as unhelpful as participant 12 (male, aged 21)

For peer review only

explained, '*I just think it's a constant reminder that I'm different. So that's why I don't really like them (reminders).'*

The other six people formed a sub-group of 'occasional' resistors. They were all men and mainly aged in their mid thirties to mid fifties. They were not bothered about people knowing about their XP but had gaps in their photoprotection routines, often going outside for short periods without sunscreen. This appeared to partly reflect perceptions of low personal risk if they had lived 20 or 30 years without major problems. There was also a view that life is uncertain and that exposure to UVR is just one of many risks they might encounter and so not a priority. They therefore preferred to *'live for today' and* have '*a happy life despite XP*.' As participant 20 (male, age 55) explained:
'I don't automatically put on sunscreen everyday even though I know I must….there's no downsides to sunscreen, so it's just time or not thinking about it or being too busy, or thinking no I can't be bothered.' He later explained, *'I would rather do things that give me pleasure in life rather than get overly* anxious *about the ransom to the possible outcomes of not taking enough care.*' Some people referred to specific personal experiences as shaping these views, such as a close relative dying unexpectedly at a young age. Other reasons given for resisting rigorous photoprotection was a questioning of the effectiveness of sunscreen or of photoprotection generally, especially if they had lesions on parts of the body normally covered.

All participants in the 'resistant' category thus emphasized the importance of leading a normal life but differed in whether the major influence on their behaviours arose from feelings of unacceptable differentness, or from perceptions of personal UVR risk being outweighed by other life priorities and risks.

3. Integrated photoprotection This category comprised 5 men and 5 women who represented the full age range from 16 to 63 years, with 4 'burners' and 6 'non-burners', All currently accepted the need to undertake photoprotection, although a few people described struggling initially as the demands of photoprotection made them feel like a 'sick' person. However, over time these practices had become *'automatic'*, '*habitual*' and *'just second nature'*. Photoprotection was therefore not currently viewed as a major practical or emotional burden leading to a 'non-normal' life and illness identity but described as '*just a part of life … It's just habit now, a routine, what I do….It's just second nature I think for me' (Participant* 19, male, age 18). Similarly photoprotection was refererred to as '..*no hardship to do because I just do it as part of my daily routine*' (Participant 16, female, age 63). These

For peer review only

participants generally felt comfortable and confident in their photoprotection routines and currently did not worry much about the risks of UVR.

This accommodation to photoprotection was described as occurring in one of two ways. A group of younger people diagnosed in childhood had adapted by spending most of their time indoors and now preferred this. For example, participant 19 (male, 18 yrs) was diagnosed when 9 years old but began to protect much earlier. Currently his routine was to set off for work at 6am when dark and his father collected him later by car. Otherwise he was mainly in the house and preferred being indoors where he played with the computer. Participant 01 (female, aged 45) also described her need to keep out of the sun as fitting quite well with her life because she was at home most of the day looking after children.

Others emphasized how they accommodated the requirements of photoprotection without withdrawing indoors. For younger people this was often facilitated by friends or family members who accepted their need to avoid UVR and adjusted activities to ensure that they were not made to feel different or excluded.
'….especially when we're going out and there's a place that's not safe and then they all understand we probably need to move somewhere else and not do what everyone wants to do, which is to enjoy the sunlight.' (Participant 09, male, age 38)

Family members often organized activities when the sun was low, encouraged indoor sports and going on holiday in the UK rather than to hotter countries. Similarly, partners might adapt their roles, as participant 10 (male, age 39) explained:
'I'm the one who would normally be staying in the car (with window film)*….and the missus would actually go into the shops, into the street to find whatever we need to get.'* ,,,,,,,,,,,,,,,
'My wife tends to do the outdoor activities with the kids, and I do the indoors.'

For older people their accommodation of photoprotection had often become easier through their long-standing experience of XP. As participant 16 (female, age 63 yrs) commented, *'Because I'm this great age now, I'm used to it….it's just part of my daily routine now…..the only time it impacts is if I'm invited to an outdoor event and either I don't go or wear a hat and stick to the shade….but that's the only time it impacts you. But I'm used to it now' (Laughter*). For older people the personal social costs of photoprotection was also often facilitated by changes in what they expected and wished to do, as participant 21 (male, aged 62) explained: *When younger I wanted to sunbathe and go on holidays with friends to hot countries but as the years went by I got used to it. Now it doesn't bother me.'*

For peer review only

Older people who had integrated photoprotection in their lives also described rejecting any stigma associated with photoprotection, with any negative views and comments described as *'other people's problem, not my problem'.*

People who had integrated photoprotection into their lives described feeling confident that their routines provided adequate personal protection. However they sometimes acknowledged scope for improvement and may sometimes have not been aware when that there practices were not sufficiently protective.

Credibility and triangulation.

Findings of different responses to photoprotection advice were presented and discussed with the four active members of the patient and public involvement team. They endorsed the findings with a minor revision regarding a subgroup that was adopted.

The two discussion groups with clinical specialists indicated that they regarded the three emergent categories as fitting with their clinical experience. They also viewed the data as helpful in increasing their understanding of the range of psychosocial meanings and contextual influences on photoprotection behaviour.

Insert Table 1: Clinic assessment of photoprotection score for three behavioural categories

Patient data for a 13 -item measure of photoprotection that is routinely used by clinic nurses to assess adherence with photoprotection[22] was examined following completion of data analysis so as not to influence how we interpreted the interviews and categorised participants. This recorded very high photoprotection behaviour scores for the 'dominated' group, whereas the 'resistors' had the lowest mean score with all scoring 7 or less apart from one participant (no.8, male, 55 years) whose score was higher as a result of having dermagard on windows at home and in the car, although behavioural aspects were in line with other resistors. Overall the 'integrated' group had a moderate score, although half had a low score indicating that their routines were often not sufficiently rigorous (Table 1).

Discussion

Three broad groups of responses to photoprotection were identified. One group, termed *'integrated'* represent what is often regarded as the ideal form of adjustment among people

For peer review only

with a chronic condition and represent the final stage of the self-management pathway in chronic illness. This stage is often referred to as 'normalization'. This term sometimes emphasizes psychological aspects in terms of bracketing off the impact of illness so that its effects on the persons' identity are relatively slight, [24] or may refer to the incorporating of illness or treatment regimens into what is viewed as a normal life [25.] Patients categorized as 'integrated' displayed both features of normalization; they no longer felt concerned about possible negative responses and their photoprotection had moved from a deliberative to an automatic and habitual process and was now a routine part of their life. This successful adaptation was accompanied by photoprotection practices they felt comfortable and confident with, although participants' accounts and adherence scores indicated that they did not necessarily achieve the ideal in terms of rigorous photoprotection and had a more moderate level of photoprotection. In contrast those 'dominated' by the requirements of photoprotection achieved high levels of UVR protection but at a high cost in terms of activity restrictions and emotional distress and resulted in what Sanderson and colleagues in their study of rheumatoid arthritis referred to as a 'disrupted normality'.[26] However, for XP this disruption largely occurred through the visibility and demands of protective measures rather than through overwhelming pain or other physical symptoms. A third group of 'resistors' did not aim to normalize photoprotection, with the 'strong' resistors engaging in what Sanderson et al [26] referred to as 'struggling for normality' at all costs, possibly trading short-term social normality for increased clinical risks and reduced normality in the longer term. This reflects descriptions of 'denial' of an illness identity and responses to stigma in relation to a range of chronic conditions, particularly among adolescents and young adults. [27-29] "Occasional' resistors partly normalized photoprotection in their life but also questioned its personal priority for them and engaged in short periods with reduced protection, often through not bothering to apply sunscreen.

This study thus identifies some similarity in the responses of people with XP and with those identified for other chronic diseases. It is also notable that the group 'dominated' by photoprotection and who had the highest adherence scores experienced considerable psychosocial disruption, whereas those who had achieved greater psychosocial normalization disclosed that they did not necessarily do all they could in terms of photoprotection and had lower photoprotection scores. It was also clear that some apparently 'irrational' behaviour, particularly among the 'resistant' group had their own rationality when considered in terms of participants' accounts of their decision-making. Employing Horne and colleagues 'Necessity-concerns' framework[30] developed to explain medicines use it was clear that photoprotection involved balancing the perceived necessity of photoprotection to reduce UVR risks with concerns about the impacts of photoprotection

on activities, feelings of stigma and emotional distress. These concerns were of particular significance for the group of 'strong resistors'. This may reflect their age and life stage, personality and general orientation to risk taking, although these aspects were not investigated. More broadly, the data indicate that changes in either perceived necessity of photoprotection or concerns about the impacts of photoprotection could lead to shifts in behavior.

The study interviews focused on individuals' current perceptions and behaviours in terms of accommodating and coping with requirements for photoprotection. However, a few participants described transitions. One transition was from 'resisting' to 'dominated' that was triggered by greater perceived necessity through their experience of a skin cancer. There was also evidence of a shift towards 'integration' which appeared to be influenced by increasing age, greater resilience and changing social expectations. However triggers and processes of change between different behavioural responses require detailed study with a longitudinal design. There are also questions of the influence of early childhood experiences including feelings of stigma and the influence of particular family management practices including the emphasis families normalization on the photoprotection behaviours of young adults.[31,32] A further key influence may be in attitudes to health risk which has been shown to result in differences in preventive health risk behavior and treatment preferences.[33] Researching these issues would contribute to what Moss-Morris[34] noted is a need for a more adequate understanding and theory of adaptation to chronic illness, while Leventhal and colleagues[35] recommended that greater attention should be given to the dynamic processes involved in coping with chronic illness and key transitions, including transitions in relation to adherence.

Study limitations. The study of a rare disease involves a number of challenges.[36] These include the necessarily limited study population that requires particular attention to ensuring confidentiality and anonymity and risks of considerable respondent burden. For example, it was not feasible to undertake further follow up interviews to explore emerging issues in greater depth or undertake respondent validation as this patient sample were also participating in other phase 1 studies and will be invited to participate in the subsequent trial. A further limitation is that clinic attenders with neurodegeneration, which occurs in around a quarter of people with XP,[9] were excluded as they are likely to face considerably different issues to people without neurodegeneration and thus require a separate study.

Informing behavioural interventions This qualitative study complements the quantitative aspects of the phase 1 programme and provides greater depth and richness of data to

For peer review only

explain the 'why', 'what' and 'how' questions. For example, why participants went out in the sun without full protection, what they felt were the impacts of photoprotection on their lives, whether and what they disclosed to friends and how they responded to questions about their appearance.

These findings will inform the design of the intervention in a number of ways. This includes identifying people *'dominated'* by photoprotection who already have a very high level of adherence and for whom the intervention would therefore not be appropriate. However, their accounts of emotional distress identified the potential risks associated with high levels of photoprotection and indicates that emotional well-being will need to be probed in the intervention sessions and responded to for individuals as appropriate.

The study identified people 'who were *resistant*' to photoprotection as having few established routines and most likely to experience a constellation of barriers to photoprotection, while those categorized as *'integrated'* shared some of these barriers. This indicates that the intervention should include both generic components and components personalized to the specific barriers relevant to each person with the aim of changing individuals' necessity-concerns balance. Generic components might include developing positive 'habits' that refers to a situation in which a stimulus (e.g. going outside) generates an impulse to act as a result of a learned stimulus response, with a positive cue-action link leading to protective behaviours.[37] Habit formation training could involve action planning and the use of environmental cues and prompts (e.g. intervention text message) to instigate the specific photoprotection behaviour. The components to address specific needs and barriers could include strategies to enhance acceptance and willingness to photoprotect as well as strategies to reduce felt stigma by managing appearance concerns and to facilitate disclosure. Patients' accounts of their lived experiences provided by the qualitative interviews could also be included in written materials to support the one-to-one sessions and overcome personal barriers to photoprotection through drawing on the direct experience of other patients.

Conclusions

This study goes beyond the identification of themes to examine the complexity and patterning of responses, including the influence of patients' illness experience, priorities and circumstances of their lifeworld[38] on their photoprotection behaviour and contributes to an understanding of adaptation and normalization in chronic illness. The data will also inform the content and design of individualized interventions to address both motivational and

volitional barriers to photoprotection of people with XP and may also be of value for other conditions with high risks of skin cancer.

For peer review only

Acknowledgements

We are most grateful Lesley Foster who was instrumental in recruitment and facilitating the interviews. We also thank the clinical specialist XP team at St Thomas' Hospital for helpful discussions and specialist nurses Tanya Henshaw & Sally Turner for adherence data. We greatly benefitted from input by the PPI panel (Cathy Coleman, Ben Fowler, Ros Tobin, Sandra Webb) and are most grateful to the study participants for the detailed accounts of their experiences and photoprotection practices.

Contributorship statement

MM supervised the study and was primarily responsible for study design, analysis and writing the paper. RA & JWal conducted interviews, checked transcripts and were involved in the development of project materials and interpretation of findings. RA had a major role in coding with MM & JWal contributing. JWal gained ethical approval. Both RS and JW as the Programme leads were involved in discussions throughout the research and contributed to study design. All authors critically reviewed and contributed to the final paper.

Competing interests

No conflict of interest to declare.

Funding

This article presents independent research funded by the National Institute for Health Research (NIHR) under its Programme Grants for Applied Research scheme (RP-PG-1212-20009). The views expressed are those of the authors and not necessarily those of the NHS, the NIHR or the Department of Health. NIHR had no involvement in study design, research process or manuscript preparation.

Data sharing statement

Due to concerns about anonymity in such a small rare disease group our research team has decided not to put raw data in a repository.

For peer review only

References

1. Dunbar- Jacob j, Mortimer-Stephens MK. Treatment adherence in chronic disease. *J Clin Epidemiology* 2001; S4 (Supp 1): 557-60

2. Middleton KR, Anton SD, Perri MG. Long-term adherence to behavior change. *Am J Lifestyle Med* 2013; 7(6):395-404. Doi: 10.1177/155982761348867

3. Cutler R.L., Fernandez- Linos, F., Frommer, M, Benrimoj C, Garcia-Cardenas V. Economic impact of medication non-adherence by disease groups: a systematic review. *BMJ Open* 2018 Doi: 10.1136/bmj open-2017-016982

4. Vermieire E, Hearnshaw H, Van Royen P, Derekens J. Patient adherence to treatment: three decades of research. A comprehensive review. *Clinical Pharmacy Therapy* 2001; 26(5):331-342.

5. World Health Organisation, Adherence to long-term therapies: Evidence for action. *World Health Organisation* 2003: WHO/MNC/03,01

6. Genetic Alliance, Rare disease UK: What is a rare disease? http://www.raredisease.org.uk/what-is-a-rare-disease/

7. Fassihi, H., Sethi, M., Fawcett, H. Wing, J., Chandler, N., Mohammed, S……..Lehmann, A.R. Deep phenotyping of 89 Xeroderma Pigmentosum patients reveals unexpected heterogeneity dependent on the precise molecular defect. *Proceedings of National Academy of Science USA*, 2016;113(9): E1236-45. doi: 10.1073/pnas.1519444113.

8. Kleijer WJ, Laugel V, Berneburg M, Nardo T, Fawcett H, Gratchev A, Jaspers NG, Sarasin A, Stefanini M, Lehmann AR.Incidence of DNA repair deficiency disorders in western Europe: Xeroderma pigmentosum, Cockayne syndrome and trichothiodystrophy. *DNA Repair* 2008; 7: 744–750.

9. Bradford, P.T., Goldstein, A.M., Tamura, D., Khan, S.G., Veda, T., Boyle, J…… Kraemer KH. Cancer and neurologic degeneration in xeroderma pigmentosum: long term follow-up characterises the role of DNA repair. *Journal of Medical Genetics* 2011; 48 (3):168-176.

10. Sethi, M., Lehmann, A.R., Fawcett, H., Stefanini, M., Jasprrs, N., Mullard, K., Turner, S……Fassihi, H. (2013). Patients with xeroderma pigmentosum complementation groups C, E and V do not have abnormal sunburn reactions. *British Journal of Dermatology* 2013; 169(6): 1279-86.

11. Tamura, D., DiGiovanna, J.J., Khan, S.G. et al. Living with xeroderma pigmentosum: comprehensive photoprotection for highly photosensitive patients, *Photodermatology, Photoimmunology & Photomedicine* 2014; 30(2-3): 146-152.

For peer review only

12. Antov D., Hollunder, M., Schliemann, S., Elsner, P. Ultraviolet exposure and protection behavior in the general population: A structured interview survey. *Dermatology* 2016; 232:11-16.

13. Diao DY, Lee TK. Sun protective behaviours in populations at high risk of skin cancer. *Psychol Res Behav Manag* 2014; 7: 9–18.

14. Skiveren J, Mortensen EL, Haedersfdal M. Sun protective behavior in renal transplant recipients. A qualitative study based on individual interviews and the Health Belief Model. *J Dermatol Treat* 2010; 21:331-336

15. Fassihi H. Personal communication.

16. Walburn J, Sarkany, R., Norton, S., Foster, L., Morgan, M., Sainsbury, K., …..Weinman ,J. An investigation of the predictors of photoprotection and UVR dose to the face in patients with XP: a protocol using observational mixed methods. *BMJ Open*, 2017, 7(8); e018364. Doi:10.1136/bmjopen-2017-018364.

17. Bartholomew, K., Parcel, G.S., & Kok ,G. Intervention mapping: A process for developing theory – and evidence-based health education programs. *Health Education & Behaviour 1998;* 25(5): 545-563.

18. J. Ritchie & J. Lewis (Eds.), *Qualitative research practice : a guide for social science students and researchers* 2003: 219-262. London: Sage.

19. O'Brien, B.C., Harris, IB, Beckman T.J., Reid, D.A., Cook, D.A. *Standards for reporting qualitative research: a synthesis of recommendations*. Acad Med.2014;89(9):1245-1251.

20. Richie, J., Spencer, L., & O'Connor, W. Carrying out qualitative analysis. In J. Ritchie & J. Lewis (Eds.), *Qualitative research practice : a guide for social science students and researchers 2003*, 219-262. London: Sage.

21. Lincoln, Y.S., Guba, E.G. *Naturalistic Inquiry* 1995. Newbury Park CA, Sage Publications.

22. Henshaw T, Turner S. Audit of ultraviolet radiation practices by patients with xeroderma pigmentosum. *Dermatological Nursing* 2016; 5 (3): 65.

23. Scambler, G. Review article: Health-related stigma. *Sociology of Health & Illness 2009;* 31(3): 441-455.doi: 10.1111/j.1467-9566.2009.01161.x

24. Bury, M. The sociology of chronic illness: a review of research and prospects. *Sociology of Health & Illness* 1991, 13(4): 451-464.

25. Deatrick, J.A., Knafl, K,A,, Murphy-Moore, C. Clarifying the concept of normalization. *Image: Journal of Nursing Scholarship*, 1999, 31(3): 209-213.

26. Sanderson, T., Calnan, M., Morris, M., Richards, P., & Hewitt, S. Shifting normalities: interactions of changing conceptions of a normal life and the normalization of symptoms in rheumatoid arthritis. *Sociology of Health & Illness* 2011; 53(4):618-623.

For peer review only

27. Johnson, H.M., Warner, R.c., LaMantia, J.N., Bowers, B.J. ‘I have to live my life like I’m old.’ Young adults perspectives on managing hypertension: a multi-centre qualitative study. *BMC Family Practice* 2016, Doi:10.1.1186/s 12875-016—428-9.

28. Gagani A, Gemao J, Relojo D, Pilao SJ. The stages of denial and acceptance among patients with chronic kidney disease. *J Innovation in Psychology*, *Education and Didactics*. 2016, 20(2): 113-124.

29. Oris L, Rassart J, Prikken S, Verschueren M, Goubert L, Moons P, Berg CA, Weets I and Luyckx K. Ilness identity in adolescents and emerging adults with type 1 diabetes: Introducing the illness identity questionnaire. *Diabetes Care* 2016 May; 39(5): 757-763. Doi.org/10.2337/dc15-2559.

30. Horne, R., Chapman, S.C.E., Parham, R., Freemantle, N., Forbes, A. & Cooper, V. Understanding patients’ adherence-related beliefs about medicines prescribed for long-term conditions: A meta-analytic review of the Necessity-Concerns framework. *Plos One* 2013; 8(12) :e80633.

31. Anderson, R., Walburn, J., Morgan, M. Experiences of stigma over the lifetime of people with xeroderma pigmentosum: A qualitative interview study in the United Kingdom. *Journal of Health Psychology* 2017, Doi: 10.1177/1359105317714643

32. Knafl, K. A., & Deatrick, J. A. (1986). How families manage chronic conditions: An analysis of the concept of normalization. *Research in Nursing & Health, 9*(3), 215-222. doi:10.1002/nur.4770090306

33. Ferrer RA, Klein WMP. Risk perception and health behavior. *Current Opinion In Psychology* 2015, 5:85-89. DOI: org/10.1016/j.copsyc.2015.03.012

34. Moss-Morris R. Adjusting to chronic illness: Time for a unified theory. British *Journal of Health Psychology* 2013; 13, 8(4): 681-686. doi.org/10.1111/bjhp.12072

35. Leventhal, H., Phillips L. A. & Burns E. The Common-Sense Model of Self-Regulation (CSM): a dynamic framework for understanding illness self-management. *Journal of Behavioural Medicine* , 2016,39, 933-946. doi 10.1007/s10865-016-9782-8.

36. Sainsbury, K., Walburn, J., Araujo-Soares, V., Weinman. J. Challenges and proposed solutions for formative research to inform systematic intervention development in rare and unstudied conditions: The case example of Xeroderma Pigmentosum. *British Journal of Health Psycholgy* 2017; 23: 229-237.

37. Gardner B, Lally P, Wardle J. Making health habitual: the psychology of ‘habit – formation’ and general practice. Br J Gen Pract 2012, 62(605): 664-666.

38. Mishler, E. ‘Viewpoint: critical perspectives on the biomedical model’. In Mishler, E., Amara Singham, L., Hauser, S., Liem, S., Osherson, R. and Waxler, N. (eds) *Social Contexts of Health, Illness and Patient Care*. Cambridge: Cambridge University Press, 1981.

For peer review only

Table 1. Clinic assessment of photoprotection protection score [(a)] for three behavioural categories

Behavioural categories	Range	Mean protection score (SD)	Protection score rating [(a)]
'Dominated'	16-20	18.25 (2.06)	High
''Resistant	3-14	6.18 (3.25)	Low
'Integrating'	7-15 [(b)]	9.90 (3.00)	Moderate

(a) Based on Ultraviolet Radiation Protection Measure applied by specialist nurses (Henshaw & Turner, 2016).

(b) Scores out of 20 based on 13 items covering 5 areas:
1.Window film 2. Sunscreen 3. Face visor or Glasses/UVR eye protection/Face buff/Hat 4. Hand protection 5. Arms/legs covered.
High score = 15-20, Moderate score = 9-14, Low score = 0-8

The score does not take account of UVR protection with heavy curtains or blinds, with only dermagard window film at home receiving a score (100% day protected = 6;
50% day=3). This reduced the score for people who spent most of their time indoors with curtains or blinds pulled.

For peer review only

Box 1. Behavioural responses to photoprotection among people with XP

Dominated by demands of photoprotection: The major driver for rigorous photoprotection was a high fear of UVR related risks. Although concerned about the impacts of photoprotection on activities and experiencing emotional distress, these did not lead to reduced photoprotection.

Resistant to photoprotection: The major driver was the desire to live a normal life which conflicted with rigorous photoprotection.

For a group of predominately younger adults their desire to distance themselves from a stigmatized identity and be accepted as normal by their peer group outweighed concerns about the clinical risks of UVR. This led to 'strong' resistance in terms of concealing their condition by undertaking fairly limited photoprotection and often not disclosing their condition to others.

For a group of mainly older adults the requirements for photoprotection had no significant impact on self-identity or disclosure but they questioned the personal risks of UVR, its perceived efficacy and the priority given to other things in life. This led to 'occasional' resistance with gaps in their photoprotection, especially going out for short periods without sunscreen.

Integrated photoprotection: UVR protection integrated into normal life and not perceived as disruptive to activities or an emotional burden. Some achieved this by largely staying indoors which they were used to from childhood and was their preferred life style. Others lived a more usual life and were assisted in this by the co-operation of friends/family and among older people by the changing values, lifestyles and expectations of older age. Many participants although confident in their photoprotection also acknowledged scope for improving their routines.

For peer review only

Supplementary Table 1: Topics and examples of open questions from interview guide

---

Introduction

Explanation that this is the first time research has been done in XP from the patients perspective and we want to learn more about the day to day experiences and challenges of living with XP from your personal point of view. We are having this conversation to find out what it is like living with XP. We are interested in your views and understanding XP from your perspective. There are no right or wrong answers.

The interview is confidential and when we write up the study any quotations that we use will be anonymous and will not be linked to your name. The staff at the XP clinic will not hear your interview and will not know what you have said.

Taking part in the interview is voluntary - you can stop at any time. If you want to stop just let me know. If you want to take a break, just let me know. You can take your time to think about what you want to say – there is no rush.

In the information leaflet we explained that we would like to record the conversation. Is this still OK with you? This is to help us with the analysis.

Topics and examples of questions

1. Participants personal story of their XP
When were you first aware of having a skin problem? How was it diagnosed? What was your understanding of XP at that time?
What do you think are the risks of XP for you? How much does this bother you?
For how long have you been attending the specialist clinic at St Thomas' Hospital? Has this been helpful?

2. General views of photoprotection
How important is photoprotection for you? Do you think it is important in all seasons including winter and on cloudy days?
What are any particular downsides of photoprotection for you?

3. Photoprotection practices
How important is sunscreen for you? How regularly do you use sunscreen?
Do you go out without using? Are there any problems with it?

Do you make special efforts to wear clothing to cover up including gloves, long sleeves, long trousers and hats or hoodie? Glasses? Is this protection easy or are there problems with having to cover up like this?

Do you wear a visor? Is it comfortable? How important to use? If not wear currently -have you ever worn one? Why did you stop?

Do you avoid going out when sunny? How does this restrict your life?
Do you do any UV protection when inside?
Have there been any changes in your photoprotection over time?

4. Psychosocial burden of XP/photoprotection

For peer review only

What are the main ways having XP affects your activities, such as work/school/leisure activities?

What are your biggest worries about having XP?
Does having XP affect you emotionally?

5. Support by family and friends
Do other people understand/support you? In what ways? How important/helpful is this?

Do you worry about what others think about you?
Do you think others respond to you differently because of your XP?
Have you had any experiences that have made you feel this way? How do you cope with this?

For peer review only

Supplementary Table 2: **Response to photoprotection by age and gender**

	Study no.	Age (yrs)	Gender (M/F)
'Dominated'	7	27	M
	13	28	M
	14	21	M
	22	26	M
'Resistant'	3	37	M
	4	36	M
	5	26	F
	6	20	M
	10	39	M
	12	21	M
	15	34	M
	18	31	F
	20	55	M
	23	55	M
	24	28	F
'Integrated'	1	45	F
	2	35	F
	8	63	M
	9	38	M
	11	16	M
	16	63	F
	17	62	F
	19	18	M
	21	62	M
	25	55	F

For peer review only

Additional Table: Checklist

Standards for Reporting Qualitative Research (SRQR)*

http://www.equator-network.org/reporting-guidelines/srqr/

Title and abstract

	Page/line no(s).
**Title** - Concise description of the nature and topic of the study Identifying the study as qualitative or indicating the approach (e.g., ethnography, grounded theory) or data collection methods (e.g., interview, focus group) is recommended	1
**Abstract** - Summary of key elements of the study using the abstract format of the intended publication; typically includes background, purpose, methods, results, and conclusions	2

Introduction

**Problem formulation** - Description and significance of the problem/phenomenon studied; review of relevant theory and empirical work; problem statement	4
**Purpose or research questio**n - Purpose of the study and specific objectives or questions	5

Methods

**Qualitative approach and research paradigm** - Qualitative approach (e.g., ethnography, grounded theory, case study, phenomenology, narrative research) and guiding theory if appropriate; identifying the research paradigm (e.g., postpositivist, constructivist/ interpretivist) is also recommended; rationale**	5
**Researcher characteristics and reflexivity** - Researchers' characteristics that may influence the research, including personal attributes, qualifications/experience, relationship with participants, assumptions, and/or presuppositions; potential or actual interaction between researchers' characteristics and the research questions, approach, methods, results, and/or transferability	6 (paras 4 & 5)
**Context** - Setting/site and salient contextual factors; rationale**	
**Sampling strategy** - How and why research participants, documents, or events were selected; criteria for deciding when no further sampling was necessary (e.g., sampling saturation); rationale**	6 (para3) 7 (para 3)
**Ethical issues pertaining to human subjects** - Documentation of approval by an appropriate ethics review board and participant consent, or explanation for lack thereof; other confidentiality and data security issues	25, 18 (para 3)
**Data collection methods** - Types of data collected; details of data collection procedures including (as appropriate) start and stop dates of data collection and analysis, iterative process, triangulation of sources/methods, and modification of procedures in response to evolving study findings; rationale**	6-8

For peer review only - http://bmjopen.bmj.com/site/about/guidelines.xhtml

**Data collection instruments and technologies** - Description of instruments (e.g., interview guides, questionnaires) and devices (e.g., audio recorders) used for data collection; if/how the instrument(s) changed over the course of the study	p.7 para 2 & Supp. Table 1
**Units of study** - Number and relevant characteristics of participants, documents, or events included in the study; level of participation (could be reported in results)	8 para 3&4
**Data processing** - Methods for processing data prior to and during analysis, including transcription, data entry, data management and security, verification of data integrity, data coding, and anonymization/de-identification of excerpts	7-8
**Data analysis** - Process by which inferences, themes, etc., were identified and developed, including the researchers involved in data analysis; usually references a specific paradigm or approach; rationale**	7-8
**Techniques to enhance trustworthiness** - Techniques to enhance trustworthiness and credibility of data analysis (e.g., member checking, audit trail, triangulation); rationale**	8 para 2

Results/findings

**Synthesis and interpretation** - Main findings (e.g., interpretations, inferences, and themes); might include development of a theory or model, or integration with prior research or theory	9-16
**Links to empirical data** - Evidence (e.g., quotes, field notes, text excerpts, photographs) to substantiate analytic findings	9-16

Discussion

**Integration with prior work, implications, transferability, and contribution(s) to the field -** Short summary of main findings; explanation of how findings and conclusions connect to, support, elaborate on, or challenge conclusions of earlier scholarship; discussion of scope of application/generalizability; identification of unique contribution(s) to scholarship in a discipline or field	16-19
**Limitations** - Trustworthiness and limitations of findings	18 para 3

Other

**Conflicts of interest** - Potential sources of influence or perceived influence on study conduct and conclusions; how these were managed	25
**Funding** - Sources of funding and other support; role of funders in data collection, interpretation, and reporting	25

For peer review only - http://bmjopen.bmj.com/site/about/guidelines.xhtml
